# Supplementary material for: Defining a good death: Perspectives of patients, relatives, and health care professionals in the Catalan context—A qualitative study
Source: PLoS One. 2024 Nov 27;19(11):e0312426. doi: 10.1371/journal.pone.0312426 (PMC11602040; doi:10.1371/journal.pone.0312426)
Supplement: S1 Table — (PDF) [file pone.0312426.s001.pdf]

Supporting Information S1 Table

Conceptual, theoretical, and methodological considerations of the qualitative study on good death in Catalonia

		General Considerations	Specific Considerations
**Context**			
**1.**	**Project rationale**	- Project commissioned by the General Directorate of Health Planning of the Catalan Department of Health to the Agency for Health Quality and Assessment of Catalonia (AQuAS) in the context of the Death Observatory of Catalonia.	- The Death Observatory of Catalonia was launched following a parliamentary agreement in 2019 (Motion 91/XI 2017).<br>- The Steering Group of the Death Observatory identified a gap to better manage end-of-life and death within the health system.<br>- Several health departments across Europe have worked to enhance the understanding of quality care standards for end-of-life situations and the needs of individuals facing death.<br>- The Strategic Chronic Health Plan and Intermediate-Long Care Strategic Health Programs in Catalonia agreed that conducting a qualitative study on the elements of a good death and the facilitators/barriers would be a step forward in breaking taboos around death and defining actionable improvements in health care and planning.
**2.**	**Type of study /purpose**	- A qualitative study to understand the complex phenomenon of a good death, taking into consideration the views and experiences of people with chronic health conditions, caregivers, and health and social professionals in Catalonia.	- The results of this project would provide a better understanding and knowledge transfer of needs, best practices, and improvements that could be made in the health system in the Catalan context.<br>- The results could help decision-makers at the clinical, care, management, and policy levels (governmental and non-governmental organizations) to define strategies/actions to improve standards to reach and understand the needs of people at the end of life.
**3.**	**Theoretical approach**	- Phenomenological<br>- Hermeneutical<br>- Socio-constructivist<br><br>Adapted from Hancock (2002), Pla (2002), and Berenguera et al. (2014).	- Capture the meaning of a good death and the barriers, facilitators, and unmet needs, taking into consideration common characteristics based on daily living, experiences, and feelings of participants; understand in depth the subjective definition of its meaning, which is a result of a learning and socialization process (phenomenological approach).<br><br>**-** Understand the individual experience of participants including events, and relationships while considering their social, cultural, and historical (life-course) contexts going beyond

		a description of what they manifest to reveal hidden meanings and interpret them (hermeneutical approach).<br>- Study the meaning of a good death and its barriers, facilitators, and unmet needs through the collective generation of meanings, using language and interaction to produce social meanings of this construct/phenomenon. The assumption is that people construct their reality and it can change based on more information and empowerment (socio-constructivist approach).
**4. Use of previous studies**	- An *ad hoc* literature review was carried out in PubMed / Medline, Google Scholar, and key reference institutions dedicated to the assessment of health services and their quality to define the qualitative study protocol, help in defining the sampling of participants, and for discussion of results.	- No previous theoretical framework was applied to carry out the analytical process in this qualitative study.<br>- Heterogeneity of opinions and great subjectivity were expected according to the literature review.<br>- Most relevant identified studies were carried out with caregivers, relatives, and health care professionals, and not with people with chronic conditions and/or at the end of life.<br>- Heterogeneity was expected according to the views and experiences of patients, caregivers/healthcare professionals as well as differences between rural and urban areas, and the presence or absence of a more or less complex chronic condition.
**5. Territorial context**	- The study was carried out in Catalonia, an autonomous region in the Northeast of Spain on the border with the south of France. Due to history, culture, and language, Catalonia is considered a nation and has a Catalan autonomous government.	- During 2023, according to the Catalan Official Statistics (IDESCAT) portal, Catalonia had 8,016,606 inhabitants living in 947 municipalities of its 4 provinces (Barcelona, Girona, Lleida, and Tarragona) with a distribution of more urban and rural areas near the sea or the mountains.<br>- In 2023, 25.6% of inhabitants were over 60, with a life expectancy of 83.6 years. The mortality rate for 1,000 was 9.03% (70,706 deaths). Catalonia presents the 2nd of 17 autonomous communities in Spain with the highest PIB (+3.0%).<br>- The Catalan health system is divided administratively into 9 health regions within the 4 above-mentioned provinces. The Spanish Ministry of Health and Government has transferred powers (in Spanish “competencies”) to rule the general budget and health planning priorities to each of the 17 Autonomous Communities, including Catalonia.

Goals and Research Questions		
**6. General goal(s) of the project**	- Learning about the opinion and experiences of patients, relatives, and professionals regarding what constitutes a good death, within the context of the Catalan health system.	The project includes a wider scope than the one presented in this paper with 3 specific project goals:<br>- Understanding the meaning that patients, relatives, and professionals attribute to a good death.<br>- Describing the barriers and facilitators to reaching quality standards and achieving this good death.<br>- Exploring the unmet/uncovered needs in the Catalan health system so that the population (citizens) can have a good death.
**7. Specific aim of this study**	- Understanding the meaning of a "good death" according to the relevant stakeholders in the health sector, including patients, relatives, and professionals in Catalonia.	- The scope of the present paper is to address the aim of the study.
**8. Research question(s) of this study**	- What is the meaning of a good death for a selection of people living in Catalonia with a chronic health condition, a relative of a patient with a chronic health condition, or a relative who has died, and for multidisciplinary health professionals?	- How do patients, relatives, and professionals understand what a good death is in the Catalan context and in general terms?<br>- What common elements does a good death include for different participant profiles, such as patients, relatives, or professionals?<br>- What differences can be seen in the opinions of what is a good death for different participant profiles?
**Ethical Considerations**		
**9. Ethical committee**	- The protocol of the present project was approved by a reference Research Biomedical Centre of Catalonia before starting.	- The research protocol, guidelines, informed consent forms, circuits to recruit and interview patients, relatives, and participation of healthcare professionals was approved by the Ethical Committee IDIAP Jordi Gol (Jordi Gol i Gurina University Institute for Research in Primary Health Care Foundation [IDIAPJGol, 21/251-P, 24 November 2022]).
**10. Forms and data management**	- The participants signed an informed consent to take part in the study and to record interviews by audio or focus group discussions by video/audio.	- A referent professional from participant Patient Associations and Health Centres was in charge of explaining the project and the specific study to participants.<br>- All interviews and focus groups discussions were recorded and kept confidentially, following the data protection regulations of the Catalan Health Authorities and the European Commission.

Methodological Considerations

Phase 1

**11. Techniques applied**	- Semi-structured in-depth interviews	- Predefined interview guidelines.<br>- A selection of photos of the nature was used to stimulate conversation further and help participants project what a good death meant to them.<br>- Two hours (between 90 and 120 minutes) in the home place of participants or the institution, they were cared for (intermediate-long care setting).<br>- Approach of researchers: EMIC, observers who listen externally.
**12. Sampling method and design**	- Intentional, reasoned, and stratified sampling<br><br>Adapted from Vázquez (2006).	- The research team and the Steering Group of the Death Observatory of Catalonia defined the sampling approach of patients and relatives according to main stratification variables: presence of chronic condition and being a patient or close relative; people with chronic conditions could be in an advanced stage near the end of life and/or death have complex condition(s), have chronic condition(s) but not advanced or complex. In this case, be a person over 65; in the case of relatives, they had to have a close relative with a chronic condition or a relative that had already died.<br>- Other variables for the stratification were adult participants from 18 years of age, gender, region/provinces in Catalonia where they lived, different specific chronic conditions (e.g., dementia, cancer, ALS with or without comorbidities), different socioeconomic and education backgrounds, and rural/urban contexts.<br>- Participants were recruited through patient associations and Health Centres (primary care centres and intermediate-long care hospitals).

**13. Recruitment of participants**	- Coordination by AQuAS with the support of patient associations and Steering Group.	- If patients and relatives met the inclusion criteria and were willing to participate, once they signed the informed consent, they were included in the study. Participating organizations sent forms to the coordinator (main researcher) with an additional form containing contact information and health status/profile for sampling and recruitment purposes.<br>- The interviewer contacted each participant to arrange an interview at their home; if they were institutionalized, in the health centre.
**Phase 2**		
**14. Techniques applied**	- Focus group discussions	- Predefined interview guidelines.<br>- During the beginning of the session a selection of photos of the nature was shown to participants in order to choose the one that represented the best what a good death meant to them and start the discussion. Also main insights from the first phase for discussion and addition.<br>- Two hours (between 2 - 2.5 hours) online using the AQuAS corporate Zoom application.<br>- In Spain, we understand a focus group discussion technique as a more open discussion on selected topics (predefined in a semi-structured guideline); it also includes multidisciplinary profiles of participants with diverse backgrounds leading to a deeper understanding of meanings, opinions, and experiences while generating collective knowledge on the topic of interest.<br>- Approach of researchers: EMIC, observers who listen from an external perspective.
**15. Sampling method and design**	- Planned, reasoned, and stratified sampling<br><br>Adapted from Vázquez (2006).	- The research team and the Steering Group of the Death Observatory of Catalonia agreed on the sampling approach.<br>- Recruiting patients and relatives proved challenging due to bioethical considerations and the complex taboos surrounding death, as well as the necessity for engaging in deep discussions to share opinions and experiences.<br>- In order to complement the sample of patients and relatives finally recruited (e.g., further vision of rural areas), professionals from patient associations were recruited, and professionals working in the health sector as healthcare professionals, managers in health policy at regional/local levels, and/or representatives from teaching and scientific societies were selected.<br>- From rural/urban areas across the 4 provinces in Catalonia, professionals working for different health providers in different health areas (primary care, intermediate care, specialized acute care, emergency care facilities), social services, regional/health planning, and disciplines (nursing, psychology, medicine from different specializations, such as social work, health economics, management), together with a balance of gender and age groups.

**16. Participants and recruitment**		- The professionals were selected through a literature review, the Steering Committee of the Death Observatory, aligned scientific societies, patient associations, or snowball effect. If they agreed to participate and be recorded, they signed by e-mail, and the research team sent a schedule and link to a Zoom meeting.
**Analyses**		
**17. Type of analyses**	- Thematic content analysis<br>- Discourse analysis<br><br>Adapted from Braun and Clarke (2006), Amezcua and Galvez (2002), Berenguera et al. (2014).	- The thematic content analysis provides a flexible and creative analytical process that allows for identifying, analysing, and reporting patterns (themes) within data. It emphasizes the manifest dimension of language. It starts from a fragmentation of the text and then integrates the parts to reach a global understanding of the phenomenon.<br>- The discourse analysis helps establish "why what is said is said." We carried out this analysis to understand the global and contextualized meaning of the text. Only later, the overall interpretation of the data is analysed in detail and in parts.
**18. Step in the analytical process**	Steps according to an applied analytical process followed in the study and described after their conceptualization, adapted from authors Braun and Clarke (2006), Berenguera et al. (2014), Amezcua, and Galvez (2002).	Step one:<br>- Revision of fieldwork notes, photographs, and key insights discussed among researchers during fieldwork.<br>- Transcription of interviews.<br>- Reading a selection of transcriptions to become familiar with texts and narratives.<br><br>Step two:<br>- Reading transcriptions to find the contents and patterns of meanings (themes) of what is a good death.<br>-Backward and forward reading to deepen these patterns, selecting words and relevant data, making a balance of frequency/relevance to support the preliminary coding and themes, following the research questions.<br><br>Step three:<br>- Checking the themes according to the code extracts among the analysed interviews.<br>- Preliminary discussion of themes and main insights with the research team.<br>- Revision of themes/categories and continued reading of subsequent interviews.<br><br>Step four:<br>- Discussion of the main insights from interviews with the Steering Committee.

		- Discussion of the main insights from interviews with participants in focus group discussions aims to stimulate debate and explore additional elements of a good death.<br><br>Step five:<br>- Revision of impressions and insights after each focus group discussion.<br>- Transcription of focus group discussions.<br>- Reading transcriptions to find additional patterns (themes) of what a good death is.<br>- Backward and forward reading to deepen in these patterns, selecting words and relevant data, making a balance of frequency/relevance to support the preliminary coding and themes, and following research questions.<br><br>Step six:<br>- Triangulation of insights and final thematic and discourse analysis to find patterns according to the participant profiles and interactions among narratives, taking into account potential beliefs and views.<br><br>Step seven:<br>- Selection of verbatims, followed by the final analysis of this selection, connecting the analysis back to the research question and existing literature, and ending with the production and publication of a technical report.<br>- Reanalysis of the narratives, insights from technical report and appendix document with the narratives.
**Triangulation**	- Participant profiles<br>- Techniques<br>- Theoretical-methodological approach<br>- Analysts	- Participant profiles: voices and opinions of patients, relatives, and professionals.<br>- Techniques: semi-structured interviews and discussion groups with narrative and visual (images/photos) sources of data.<br>- Theoretical methodological approaches: phenomenological, hermeneutical, and partly socio-constructivist.<br>- Analysts: two researchers with different perspectives and backgrounds.<br>- Further review of insights and discussion with the research team and the Steering Group with different backgrounds (disciplines, workplaces and positions).

Reflexivity		
**Reflexivity**	Multidisciplinary research team, definition of a Steering Group	VSS is a sociologist with a diploma in Health Studies, a PhD in Medicine (biomedical research and public health), and an expert in health service research and quality in healthcare evaluation. She has been working with the mixt methods approach for more than 25 years in complex health phenomena to create knowledge transfer tools in governmental institutions.<br>LLF is a psychologist, MSc. In Clinical Psychology. Independent freelance consultant and professor of qualitative research and consumer psychology at ELISAVA. She has been applying qualitative research for more than 25 years, mainly in the private sector.<br>MEC is a medical doctor specializing in preventive medicine and has a PhD in Medicine (biomedical research and public health). Director of the area of Accomplishment and Quality Area and responsible for the Health Observatories, including the Death Observatory.<br>AGA is a health economist, MPH and has a PhD in Medicine (biomedical research and public health), expert in incorporating decision-making tools in public policies and health services. She is responsible for the Central of Economic Balances of Catalonia and initially for the Death Observatory.<br>- There has been a constant revision of main themes and insights, and discussion among a multidisciplinary research team and a Steering Group to understand patterns, meanings, and concepts, taking into account a functionalist perspective together with a critical perspective.<br>- The Steering Group includes key representatives from aligned scientific societies (nursing, palliative care, clinical psychology, social services, geriatrics, primary care), non-governmental organizations that represent the voices of people with different health conditions and end-of-life needs, bioethical organizations, and health planning in Catalonia.
