# Supplementary material for: Defining a good death: Perspectives of patients, relatives, and health care professionals in the Catalan context—A qualitative study
Source: PLoS One. 2024 Nov 27;19(11):e0312426. doi: 10.1371/journal.pone.0312426 (PMC11602040; doi:10.1371/journal.pone.0312426)
Supplement: S2 Table — (PDF) [file pone.0312426.s002.pdf]

# Supporting information S2 Table

Topics raised in In-depth interviews and focus group discussion sessions with patients, relatives, and professionals in the qualitative study

| **Semi-structured in-depth interviews with patients and relatives** | **Focus group discussions with professionals** |
|---|---|
| 1. Life context | 1. Definition of a good death from a professional and/or personal perspective (*) |
| 2. Impact of their illness, aging, or the impact of their relative's illness | 2. Discussion of the definition provided by patients and relatives about what a good death is (*) |
| 3. Perception and meaning of death (*) | 3. Discussion of the facilitators and barriers to achieving this good death reported by patients and relatives (Phase 1) |
| 4. What is a good death? (*) | 4. Needs not covered by the health and social systems for achieving this good death |
| 5. Things that can help achieve this good death and things that may be difficult achieving it. Impact of COVID-19 | 5. Practical aspects or actions that could be applied in the health and social systems to help achieve this good death |
| 6. Things the public health and social systems can do to help people achieve this good death | |

(*) The present paper supplies documentary evidence of the main findings on these topics.
