# Supplementary material for: Defining a good death: Perspectives of patients, relatives, and health care professionals in the Catalan context—A qualitative study
Source: PLoS One. 2024 Nov 27;19(11):e0312426. doi: 10.1371/journal.pone.0312426 (PMC11602040; doi:10.1371/journal.pone.0312426)
Supplement: S3 Table — (PDF) [file pone.0312426.s003.pdf]

Suporting information S3 Table

Quotes from participants that reinforce the eight core elements for facilitating a good death

**Comfort and placidity**<br>*"Something I'm very clear about is that I don't want to suffer* [...] *and the truth is that ...PADES [Palliative and End-of-Life Program] means a lot in my life. I also thought about talking to them about not wanting to suffer, because they are working with pain issues* [...] (Woman with palliative care needs & advanced chronic conditions, 26-45 years).<br>"[...] *I've spent many hours thinking, 24 hours, and precisely, it's not that I prefer death, but rather that, I prefer to avoid suffering"* (Man with complex chronic conditions, 65-79 years).<br>*"Pain doesn't kill you, you know?* [...] *What scares me is not having a solution at hand and suffocating... Seeing that I'm dying and not being able to... they might not give me anything, either to calm me down or to fall asleep or whatever, that's what scares me"* (Woman with complex chronic conditions, 65-79 years).
**Safety**<br>*"For me, dying alone… Well, alone…, with an efficient team of health professionals who administer whatever is needed to make it as fast and as painless as possible"* (Woman with complex chronic conditions, 65-79 years).<br>*"For me, with the injection [referring to what is a good death]; for me, the intravenous injection would be better, with a person, a health professional, who knows that the needle is inserted correctly and won't come out* […]*"* (Woman, over 65 years without a diagnosis of complex or advanced chronic conditions).
**Warmth**<br>*"I am afraid of dying in certain ways... For example, suffocating, and above all, dying alone... It is the fear of the unknown. However, if you have someone with you, you are no longer alone* […] (Woman with complex chronic conditions, 65-79 years).<br>*"I imagine it* [her death]*, hugging everyone... whoever wants to.* [...] *Touching each other, in contact, and then of course, suddenly I will no longer be there, in that body. They will be well prepared beforehand, OK? And they'll know what I want* […]*"* (Woman with complex chronic diseases, 65-79 years).<br>*"Your mother's* [her husband's mother; referring to her death]*… what we did was an affectionate accompaniment because it was hard for her, and what we did was to accompany her as best we could right to the end* […]*" (R*elative of a person with an advanced or chronic condition, woman, 46-64 years old).
**Harmony**<br>"[...] *I don't think about it so much for me (in death), as much for the grief it generates in my immediate environment... (...) I have a folder here that says: "When I die, look here, I have explained everything I needed to explain"... Well, my last will and*

testament, (...), photo albums that, for example, I have them, and I am already emptying them. (...) Keep in mind that I am subscribed to this, to that... So remember to stop my subscriptions […]*"* (Man with complex chronic diseases, 65-79 years).<br><br>*"There is no such thing as a "good" death, because no one has any interest in dying, unless you say, "well, I am leaving," right? Nevertheless, "good" is that... Above all, no suffering and you can, more or less, say, "okay, from now on I'm letting go, or from now on give me a jab… And I'll leave, and that's all"* (Woman with complex chronic diseases, 65-79 years).<br><br>*"A peaceful death… no confrontations with anyone… don't leave any unfinished business here in this life…* […]*. I told you that I do not want to leave an inheritance; I have sorted everything out: everything was written down and in front of a notary so that no one will fight.* [What else does "leave no unfinished business," mean?] *There are two ways of saying goodbye to people; for the WhatsApp group, I already have a note prepared, which I will send before I die if I can* […]*"* (Man with palliative care needs & advanced chronic conditions, 65-79 years).
**Intimacy**<br><br>*"What we can't allow, and what they have to fight for in the hospital, is that someone dies with the sound of TV5* [Spanish TV channel] *in the background. That is how it is! [...] It is one thing if someone dies from a heart attack* [suddenly], *but if it's a person who's in the end-of-life process... They can't be in a shared room* [referring to the moment of dying]*, or with the sound of TV5 in the background* [...]*"* (Woman with complex chronic diseases, 65-79 years).<br><br>*"Intimacy is having a private space, where all the 'external stimuli' I choose are pleasant. Maybe…, with the love of my relatives, with my relatives there, or I imagine a window to look out and see nature. I include these external stimuli in the concept of intimacy. It is also important to avoid other stimuli, such as noise, conversations of other people outside that intimate moment [...]* (Woman, social worker from the health system).
**Respect**<br><br>*"Christians and Catholics believe that later we will be with God. Therefore, (death) is not an end, but a pause on a path; […] what worries me is how it will happen, or the environment I will leave.* […] [What worries you about the "how"?] *That I won't be able to decide, that I won't decide either how or when I die" (Man with palliative care needs & advanced chronic conditions, 26-45 years).*<br><br>*"I don't want this* [referring to the way her father died]*;* [...] *I have a document that says what I want, my fear is that...* [...]*, I think: when I say enough, I mean enough; I do not want them to keep me here... My life is mine. On the one hand, I don't want to suffer: my vision is to not suffer, I don't want to suffer because I have seen suffering* [referring to loved ones]*"* (Relative of a person with an advanced or chronic condition, woman, 46-64 years old).
**Peacefulness**<br><br>*"In this house, if I'm gone, everything will remain exactly the same; that is to say, I'm already content, satisfied, I can leave in peace, I won't say: "Look, this person will need this and that person will need that"* (Man with palliative care needs & advanced chronic conditions, over 80 years old).

A rest... Tranquillity, what the Greeks called "apatheia", tranquillity of spirit... (Man with complex chronic diseases, 65-79 years).

"The ideal thing would be to die peacefully at home. Accompanied by the family, and that is all. […] A professional who applies the sedation and the rest, and that is it. [What is important for you?] *If I die, what will happen to them, right? How will they be? How will they experience it? What will happen next, right? I mean... what are the consequences of me dying in two or in 10 years?* [What would you like?] *To know that they will be completely fine, economically, physically, psychologically"* (Man with complex chronic conditions, 26-45 years).

Fulfilment

"This feeling of 'I can't leave', 'what are my children doing' [...]*, 'no, I can't leave, I can't leave them'.* [...] *Until it got to a point when I said: 'Listen, I've already done everything I could do; I have a family, it's continuing, for better or for worse, but in the end, it's no longer my concern," this is what I told my children" [...]* (Relative of a person with an advanced or chronic condition, man, 65 to 79 years old).

"I sometimes say a quote [...]: 'We were rich and we were happy, and we didn't know it', and I realize now, that I was rich and happy... Moreover, I can thank myself, my friends, and my husband, and say 'Be happy, go ahead, when you think of me...'. I can do the closure, and even more, I want to do it this way also, because I think that I also give my family and friends some help to go on with their lives with enthusiasm and optimism... The only thing I want is this: to have a death worthy of myself because I will be happy with myself. [...]*"* (Woman with palliative care needs & advanced chronic conditions, 65 to 79 years old).

Additional findings and quotes from participants that reinforce how a good death is related to a good life

The perspective of a good life and a good death

The findings from in-depth interviews conducted with people living with a chronic condition or receiving palliative care, those over 65, and their close relatives indicate that the concept of a good death (one's own or that of a relative) is closely linked to having lived a good, long life while being able to maintain a minimum quality of life. In this context, the idea of a projected "good death" refers to the idea of having enjoyed a minimum of "quality of life" during the time before death, or in the words of a participant: *"I'm pleased that you want to know (with this study) what a good death means for people, but it's pointless unless you first make sure we have the means to live a good life*[…]*"* (woman with complex chronic diseases, 65 to 79 years old).

Additional quotes from participants that reinforce this insight.

"I ask life to accept me, and […] *doctor X (name of doctor)* […] *will decide which nurse is to come to my home, here, to give me the 'little injection' and I will be transformed into a hummingbird.* […] *And…, I'll think to myself: well, I've been very lucky, I've loved a lot, I have felt very loved;* […] *my daughter is quite happy with her life, she is in (place where she lives); my sister also has a partner. It has been a good life; goodbye. [...] It is that*

simple, death is simple. If you're clear in your mind […] the only thing that worries me is that this (her illness) happened all of a sudden very quickly and I did not have the papers (advance healthcare directive, AHD)" (woman with complex chronic diseases, 65 to 79 years old).

"This is life, life ends, life comes at a time when it ends and it ends for everyone; then we must try to see it as something normal, see that it is part of our life; I refuse to see it as a horrible end, the end of a stage when everything ends. No, it is a normal, natural process that we all go through and that we must accept, and that we must accept now! We will die and that is it, full stop. [...] *Life must be lived, life must be lived intensely, without harming anyone, enjoying the little moments, enjoying your friends, your family, living with all the intensity... All this, I think that knowing how to live prepares you to know how to die... Death is another stage of life, it is another stage; and then, if you live intensely, you will die intensely, in the sense that I think: you should not dwell on the negative, sinister part of death. I do not know... I think that there are cultures that we think are less developed than us but I think are much more* [developed]*, and precisely because they experience death much more naturally than we do* [...]*"* (woman, over 65 years old without a diagnosis of complex or advanced chronic conditions).

"My motto is 'I want to die exactly as I have lived and I have lived trying to be the best I can, have the most comfortable sofa and the most comfortable mattress on my bed, and I want to die on a comfortable mattress and sleep peacefully […]. *The only thing that scares me a little is not being able to die when I want.* [...] *Some people can decide* […] *the idea I might die violently and aggressively* […] *is the only thing that terrifies me.* [...]*"* (woman, over 65 years without a diagnosis of complex or advanced chronic conditions).

"These days I've been thinking about all this, but the title you have given it, 'a good death', doesn't seem quite right to me. [...] *Really, death for me is the path that I am now taking towards death; it's the process, it's this walking, and for me, this walking is what really matters. […] I'll have a good death because at that moment I won't feel anxious; ... what I want is that the corridor or this space that I still have until I get there, this walk, should be as rewarding as possible, with a good life... With a good quality, with a good sensibility, which is what I'm doing now, doing it my way* [...]*"* (woman with palliative care needs & advanced chronic conditions, 65 to 79 years old).

"[...] *I think that it is also related to the fact that we want to die quickly, and that we don't know how to die. Therefore, of the deaths, we have known so far, the best are the quick ones; we are afraid to talk about death. Well, we are all very aware of these things..., but maybe yes, in a few years or in a time when we are more prepared for death, then yes, if we continue with the topic of euthanasia, and chronic diseases, one can decide when it is the right time and can prepare for it... So maybe we should not want to die quickly. I think it is a bit like the culture of dying badly* [...]*. On the other hand, this thing about dying quickly in the society we live in, with a life expectancy that's so long... people die very slowly; with several chronic diseases; a slow, continuous death, and it's getting closer, isn't it? So, well, first* [we need to] *help people live in the most dignified way they can in these times, but, of course, also make them aware that they're getting closer to death."* (Woman, general practitioner in primary health care).
