# Supplementary material for: Defining a good death: Perspectives of patients, relatives, and health care professionals in the Catalan context—A qualitative study
Source: PLoS One. 2024 Nov 27;19(11):e0312426. doi: 10.1371/journal.pone.0312426 (PMC11602040; doi:10.1371/journal.pone.0312426)
Supplement: S4 Table — (PDF) [file pone.0312426.s004.pdf]

Supporting Information S4 Table

Core Elements of a Good Death, Participants, Design of this Study and Other Selected Studies on the Topic

Phase in the study	Study in Catalonia	Hirai et al. 2006	Meier et al. 2016	Kinghorn et al. 2018	Krikorian et al. 2019	Gafaar et al. 2020	Wang et al. 2023
Key elements	**n=8 elements:** comfort & placidity, safety, warmth, harmony, intimacy, respect, peacefulness, fulfilment	**n=17 elements:** freedom from pain and symptoms, good family relationships, dying in favourite place/ environment, having a good relationship with the medical staff, not being a burden to others, maintaining dignity, completion of life, maintaining sense of control, hope, fighting against cancer, contributing to others, not prolonging life, preparation for death, not being aware of death, appreciating others, maintaining pride, having faith	**n=11 elements:** preferences for the dying process: pain-free status, emotional wellbeing**, family, dignity, life completion, religiosity/ spirituality, treatment preferences, quality of life, relation with health professionals	**11 capabilities of experiencing good death:** communication and acknowledgement, emotional and psychological support, spirituality and reflection, choice and autonomy, being with important people, pain and symptom control, dignity and being treated with compassion, having affairs in order and having a sense of completion	**n=5 elements:** control of pain and symptoms, feeling of closure, being able to give to others, preparation for death, still being seen as a person **Factors that influence a good death:** life circumstances, age, culture, financial issues, religion, disease	**n=7 elements:** religion and spiritual wellbeing, familial and interpersonal wellness, grief copying and emotional wellness, optimal timing, minimal suffering and burden, quality of care by formal caregivers, quality of care as informal caregivers	**n=7 elements:** being aware of cancer, pain and symptom management, dying well, being remembered after death, individual perspectives of a good death, individual behaviours leading to a good death, culture and religion
Profile of participants	- Patients<br>- Relatives<br>- Professionals	- Patients<br>- Families<br>- Professionals	- Patients<br>- Families<br>- Professionals	- Academic expert<br>- Clinical and non-clinical professionals	- Patients	- Patients<br>- Relatives<br>- Health professionals	-Patients with cancer

Study design	Qualitative (sites in Catalonia, north-eastern Spain):<br>- In-depth interviews<br>-Focus group discussion	Qualitative (sites in Japan):<br>- Semi-structured interviews<br>Quantitative:<br>- Survey; factor analysis	Systematic review of qualitative and quantitative studies (n=36)	Qualitative (sites in UK or Republic of Ireland):<br>- Semi-structured interviews	Systematic review of qualitative and quantitative studies (n=29)	Qualitative (sites in Northern Tanzania):<br>- Focus group discussions	Systematic review of qualitative studies (n=5)

** Core top elements.
