# Supplementary material for: Defining a good death: Perspectives of patients, relatives, and health care professionals in the Catalan context—A qualitative study
Source: PLoS One. 2024 Nov 27;19(11):e0312426. doi: 10.1371/journal.pone.0312426 (PMC11602040; doi:10.1371/journal.pone.0312426)
Supplement: S1 File — (PDF) [file pone.0312426.s005.pdf]

Supporting Information S1 File

Additional Insights on the Preferences for the Place to Die as a Facilitator for Achieving a "Good Death"

Main insights on the place to die as a facilitator for a "good death" and its connection to the core element of "intimacy" (see S3 Table and main results of the present paper)

The ability to choose the place to die was considered a facilitator for achieving a good death by some patients, as they expressed the desire to be in an intimate peaceful and harmonious environment. Their home is usually the preferred site; because it is considered the most intimate, especially when patients fear hospital restrictions on visiting hours might prevent their loved ones from accompanying them freely. However, hospitals offer a stronger sense of safety, particularly when managing potential clinical complications during the dying process. They also remove a burden associated with dying at home, as family members may come to associate specific locations within the house with the death("[…] every time my daughter passes by the hall, she will think 'here's where mom died'"). Additionally, hospitals simplify the logistics of transferring the body to the mortuary ("dying in the hospital is more hygienic; they don't have to come to your home to collect your corpse"). In this regard, patients, relatives, and professionals point out that the ancient custom of watching grandparents, parents, and other relatives or neighbors die, and watching over them at home, has been lost – a custom that made death familiar and natural. In a way, the modern shift toward holding vigils at the mortuary has made death feel like a more "aseptic," "denatured" event.

Regarding the ideal site for dying, some patients and relatives proposed a third option beyond the home or the hospital which to some extent offers the advantages of both and avoid the inconveniences: "the hotel where people go to die". This concept envisions a comfortable, customizable space outside the home that is properly equipped with medical care—"a hotel that isn't a real hotel, but has all the necessary facilities." In India, since 2015, there have been end-of-life hostels, and in the United Kingdom, hospices offer end-of-life assistance in spaces created for this purpose.

In any case, the space must ensure a minimum level of privacy and tranquility allowing for intimacy – shielded from outsiders and free from disruptive elements such as social media. To this end, some patients, relatives, and professionals personalize the room using sensory elements like music, scents, or certain clothes to increase the patient's perception of comfort and peace, both important for achieving a good death: "I know exactly which song I want to play when I die, "*El día que yo me muera", by Rozalén*…" "I hope I can die at my partner's house, so we can see the fantastic woods together. We have already talked about it." "For me, smell is important; I want to die in a place that smells nice." For some people, incorporating a certain ritual around their death helps them perceive it in a more natural, harmonious way reducing the sense of threat and fear.

However, if death comes sooner than expected, the patient may not have had the time to prepare everything (for example, an advance healthcare directive, a will, farewells, etc.). Professionals stress the importance of encouraging patients to reflect on these issues before their state of health deteriorates - rather than now they receive an end-of-life diagnosis - when both patients and relatives are already overwhelmed. Ideally, the goal is to die peacefully at home.

[Preference at home] *"*[...] *In addition, accompanied by my family.* […] *A professional will come to sedate me and whatever else, and that is all* […] (man with palliative care needs & advanced chronic conditions, 26 to 45 years old).

"Where? I do not know […] *I had planned to go to (name of the hospital) because I have (a relative) there and I know that she will handle my death well...* [...] *the PADES* [End-of-life Palliative Care Program] *works well. Why don't I want to be at home? Because it is more hygienic to die in a hospital. My best friend died at home and it was a drama. I was with him and I saw how they treated him when they took the body away... In a hospital, they cover you up and then take you downstairs... Yes, you want to die in peace, but seeing people crying does not give you peace.* […] *Like the shamans over there, who go to the mountain and die alone, that is the best death?* [What makes it the best death?] *Dying always hurts others. It does not hurt you, because you leave and say... "We have come this far"* (man with palliative care needs & advanced chronic conditions, 65 to 79 years old).

[What resources do you need to have a good death?] *"Being surrounded by family... being in my home* [...]. *Being at home* […]. *I am not afraid of death: when it arrives, it will arrive; the only thing that scares me is suffering"* (woman with palliative care needs & advanced chronic conditions, 65 to 79 years old).

"My partner has a house in (name of the village), which has a little forest in front of it, full of trees which is very nice. I love going there, at weekends, if I can go there, well; I want to invite the people I love... Obviously, my partner, my daughter, my sister, and then I have two close friends who I would like to come too... They will have to be brave to be there when I get the injection. [...] *Well, I imagine... that we will arrange it nicely. In the living-dining room of the house, there is a large window that overlooks this forest: to be there with a doctor and a nurse with who I have built up a good relationship.* […] *So, how do I imagine my death? I imagine it in a romantic way; I imagine it in the environment that I love, in front of nature and the trees that I love, with the three people that I love the most...* [She starts crying] *In addition, saying goodbye to them and that it has been a pleasure to know them. That is it. Then they will give me an injection here and I will pass out into the cosmos.* [...] *When I left (name of the hospital) I said: I'm giving them nine out of ten"* (woman with complex chronic diseases, 65 to 79 years old).

"For me, it is a physical process linked to health and so on; […] *I don't care if I die at home or in the hospital; maybe it's better in the hospital because I know for sure all my needs will be taken care of, but if they can do it just as well at home, and it has to be at home, that's fine, but I wouldn't mind dying in the hospital. In an operating room, I do not think so* […] *you cannot plan things: you went in with the idea of having something fixed and then you do not come out; well, I do not know, and besides I will be unconscious... At home, you leave a mark; it's where my mother died; I don't know, it's where you lived, and I've lived so many hours of my life in a hospital that I feel comforted there and I wouldn't care if I had to die there: no problem. Then, of course, you might die suddenly, in the middle of the street or wherever* […]*. (What would you prefer?) I would choose a room in a hospital and with palliative care professionals next to me in case I start the process (referring to dying).* […]*"* (woman with complex chronic diseases, 65 to 79 years old).

[Where he wants to die] *"At home; I know it's a nuisance for others and, therefore, I'd like to avoid it... If I get very old, I do not care if they take me to a nursing home. Why? Because it is a liberation for the others and that is the goal: to free them, not to be a burden for them.* [...] [The advance healthcare directive document] *I make it clear that I don't want… them to prolong my life for a few days more just for the sake of it* […]*, let me die in peace, that's it. Just that. They do not have to do extra things to prolong my*

life, which might not be life anymore; it is existence, not life... Life is when you have this capacity for enthusiasm, for a project, for joy, for mental integrity, for creativity. Existence is simply when your timeline is extended without any sense […]*."* (Man with complex chronic diseases, 65 to 79 years old).
