# Supplementary material for: Defining a good death: Perspectives of patients, relatives, and health care professionals in the Catalan context—A qualitative study
Source: PLoS One. 2024 Nov 27;19(11):e0312426. doi: 10.1371/journal.pone.0312426 (PMC11602040; doi:10.1371/journal.pone.0312426)
Supplement: Attachment — Submitted filename: PONE answers editorial team and reviewersvf.docx [file pone.0312426.s006.docx]

PONE-D-23-14275

What is a good death for patients, families and health professionals? A qualitative ethnographic and phenomenological study in the Catalan context

PLOS ONE

Dear Dr. Serra-Sutton,

Thank you for submitting your manuscript to PLOS ONE. After careful consideration, we feel that it has merit but does not fully meet PLOS ONE’s publication criteria as it currently stands. Therefore, we invite you to submit a revised version of the manuscript that addresses the points raised during the review process.

Please submit your revised manuscript by Sep 16 2023 11:59PM. If you will need more time than this to complete your revisions, please reply to this message or contact the journal office at [plosone@plos.org](mailto:plosone@plos.org). When you're ready to submit your revision, log on to <https://www.editorialmanager.com/pone/> and select the 'Submissions Needing Revision' folder to locate your manuscript file.

Please include the following items when submitting your revised manuscript:

- A rebuttal letter that responds to each point raised by the academic editor and reviewer(s). You should upload this letter as a separate file labeled 'Response to Reviewers'.
- A marked-up copy of your manuscript that highlights changes made to the original version. You should upload this as a separate file labeled 'Revised Manuscript with Track Changes'.
- An unmarked version of your revised paper without tracked changes. You should upload this as a separate file labeled 'Manuscript'.

If you would like to make changes to your financial disclosure, please include your updated statement in your cover letter. Guidelines for resubmitting your figure files are available below the reviewer comments at the end of this letter.

If applicable, we recommend that you deposit your laboratory protocols in protocols.io to enhance the reproducibility of your results. Protocols.io assigns your protocol its own identifier (DOI) so that it can be cited independently in the future. For instructions see: <https://journals.plos.org/plosone/s/submission-guidelines#loc-laboratory-protocols>. Additionally, PLOS ONE offers an option for publishing peer-reviewed Lab Protocol articles, which describe protocols hosted on protocols.io. Read more information on sharing protocols at <https://plos.org/protocols?utm_medium=editorial-email&utm_source=authorletters&utm_campaign=protocols>.

We look forward to receiving your revised manuscript.

Kind regards,

Martin Mbonye
Academic Editor
PLOS ONE

Additional Editor Comments:

Dear Dr. Serra-Sutton,

Thank you for submitting your manuscript to PLOS ONE. After careful consideration, we feel that it has merit but does not fully meet PLOS ONE’s publication criteria in its current form. Therefore, we invite you to submit a revised version of the manuscript that addresses the points raised during the review process.
The manuscript has been evaluated by two reviewers, and their comments are available below. The reviewers have raised a number of concerns that need attention. A common comment from both reviewers is on the disconnect between the introduction and the methodology which you have to address. Pay close attention to the comments on the methods section in general pointed out by both reviewers. Reviewer 2 was particular about the language editing that you need to pay attention to. Could you please revise the manuscript to carefully address the concerns raised?
Please submit your revised manuscript by Sept 15 2023 11:59PM. If you will need more time than this to complete your revisions, please reply to this message or contact the journal office at [plosone@plos.org](mailto:plosone@plos.org). When you're ready to submit your revision, log on to <https://www.editorialmanager.com/pone/> and select the 'Submissions Needing Revision' folder to locate your manuscript file.

Please include the following items when submitting your revised manuscript:
• A rebuttal letter that responds to each point raised by the academic editor and reviewer(s). You should upload this letter as a separate file labeled 'Response to Reviewers'.
• A marked-up copy of your manuscript that highlights changes made to the original version. You should upload this as a separate file labeled 'Revised Manuscript with Track Changes'.
• An unmarked version of your revised paper without tracked changes. You should upload this as a separate file labeled 'Manuscript'.
If you would like to make changes to your financial disclosure, please include your updated statement in your cover letter. Guidelines for resubmitting your figure files are available below the reviewer comments at the end of this letter.

If applicable, we recommend that you deposit your laboratory protocols in protocols.io to enhance the reproducibility of your results. Protocols.io assigns your protocol its own identifier (DOI) so that it can be cited independently in the future. For instructions see: <https://journals.plos.org/plosone/s/submission-guidelines#loc-laboratory-protocols>. Additionally, PLOS ONE offers an option for publishing peer-reviewed Lab Protocol articles, which describe protocols hosted on protocols.io. Read more information on sharing protocols at <https://plos.org/protocols?utm_medium=editorial-email&utm_source=authorletters&utm_campaign=protocols>.

We look forward to receiving your revised manuscript.

Kind regards,

Martin Mbonye
Academic Editor

**Answers to Editors and Reviewers**

Dear Dr Mbonye, the editorial team, and reviewers of PLOS ONE,

We are very grateful for your suggestions regarding our manuscrit, which have enabled us to introduce important improvements to clarify the findings and the scope of the present paper for readers of PLOS ONE.

We hope that we have responded satisfactorily to all the suggestions made by the reviewers and editorial team, and that you consider the content to be of interest to an international readership.

Please find a revised version of the manuscript with additional appendix materials, and do not hesitate to contact us if you have any further comments or suggestions.

Sincerely,

Vicky Serra-Sutton

Corresponding author

**PLOS ONE**
Note: HTML markup is below. Please do not edit.]

Journal Requirements:

When submitting your revision, we need you to address these additional requirements.

**1. Please ensure that your manuscript meets PLOS ONE's style requirements, including those for file naming. The PLOS ONE style templates can be found at**

<https://journals.plos.org/plosone/s/file?id=wjVg/PLOSOne_formatting_sample_main_body.pdf> and

<https://journals.plos.org/plosone/s/file?id=ba62/PLOSOne_formatting_sample_title_authors_affiliations.pdf>

**Answer to editorial team:** The authors have followed the style requirements of PLOS ONE.

**2. Thank you for stating the following financial disclosure:**

"The study is part of a governmental commissioned project of the General Directorate of Health of the Catalan Health Department from the Government of Catalonia (Spain) in the context of the implementation of the Death Observatory to the Agency for Quality in Healthcare and Assessment of Catalonia (AQuAS)."

At this time, please address the following queries:

a)        **Please clarify the sources of funding (financial or material support) for your study. List the grants or organizations that supported your study, including funding received from our institution.**

**Answer to editorial team:** As mentioned above, the study is part of a commissioned project but did not receive any specific funding or grant for its implementation.

b)        **State what role the funders took in the study. If the funders had no role in your study, please state: “The funders had no role in study design, data collection and analysis, decision to publish, or preparation of the manuscript.”**

**Answer to editorial team:** The commissioners had no role in study design, data collection and analysis, the decision to publish or preparation of the manuscrit. Nevertheless, the research protocol and preliminary findings were discussed with the commissioners from the Department of Health of Catalonia to increase usefulness and impact for knowledge transfer and decision-making.

c)        **If any authors received a salary from any of your funders, please state which authors and which funders.**

**Answer to editorial team:** None of the coauthors received a salary from the commissioners. Lluïsa Fernández Giner received a salary from the Agency for HealthCare Quality and Assessment of Catalonia (AQuAS) as external and independent consultant paid through a general butged of internal comissioned project linked to the Death Observatory of Catalonia (Good death study code: 0002.LE01.20.037).

d)        **If you did not receive any funding for this study, please state: “The authors received no specific funding for this work.”**

**Answer to editorial team:** The authors received no specific funding for this work.

Please include your amended statements within your cover letter; we will change the online submission form on your behalf.

3. **In your Data Availability statement, you have not specified where the minimal data set underlying the results described in your manuscript can be found.** PLOS defines a study's minimal data set as the underlying data used to reach the conclusions drawn in the manuscript and any additional data required to replicate the reported study findings in their entirety. All PLOS journals require that the minimal data set be made fully available. For more information about our data policy, please see <http://journals.plos.org/plosone/s/data-availability>.

**Answer to editorial team:** The narratives of participants and verbatims/quotes supporting the main insights of the study are freely available on the website of AQuAS, the public governmental institution dedicated to evaluating the quality of healthcare and development of decision-making tools/knowledge transfer.

For further information, please consult:

https://aquas.gencat.cat/web/.content/minisite/aquas/publicacions/2022/estudi_qualitatiu_bona_mort_annexos_aquas2022.pdf

Upon re-submitting your revised manuscript, please upload your study’s minimal underlying data set as either Supporting Information files or to a stable, public repository and include the relevant URLs, DOIs, or accession numbers within your revised cover letter. For a list of acceptable repositories, please see <http://journals.plos.org/plosone/s/data-availability#loc-recommended-repositories>. Any potentially identifying patient information must be fully anonymized.

Important: If there are ethical or legal restrictions to sharing your data publicly, please explain these restrictions in detail. Please see our guidelines for more information on what we consider unacceptable restrictions to publicly sharing data: <http://journals.plos.org/plosone/s/data-availability#loc-unacceptable-data-access-restrictions>. Note that it is not acceptable for the authors to be the sole named individuals responsible for ensuring data access.

We will update your Data Availability statement to reflect the information you provide in your cover letter.

4. **We note that you have stated that you will provide repository information for your data at acceptance.** Should your manuscript be accepted for publication, we will hold it until you provide the relevant accession numbers or DOIs necessary to access your data. If you wish to make changes to your Data Availability statement, please describe these changes in your cover letter and we will update your Data Availability statement to reflect the information you provide.

**Answer to editorial team:** Additional materials will be made available on acceptance of the manuscript in PLOS ONE.

 5. **Please include captions for your Supporting Information files at the end of your manuscript, and update any in-text citations to match accordingly**. Please see our Supporting Information guidelines for more information: <http://journals.plos.org/plosone/s/supporting-information>.

**Answer to editorial team:** Captions have now been added in appendix material.

Reviewers' comments:

Reviewer's Responses to Questions
Comments to the Author

**1. Is the manuscript technically sound, and do the data support the conclusions?**

Reviewer #1: Partly
Reviewer #2: Partly

**Answer to reviewers:** We have improved the manuscript by including additional verbatims/quotations that support the main findings and conclusions.
________________________________________

**2. Has the statistical analysis been performed appropriately and rigorously?**
Reviewer #1: I Don't Know
Reviewer #2: N/A

**Answer to reviewers:** The project applied a qualitative ethnographical and phenomenological approach; as no quantitative data were obtained, no statistical analysis was performed.

___________________________________
**3. Have the authors made all data underlying the findings in their manuscript fully available?**

The PLOS Data policy requires authors to make all data underlying the findings described in their manuscript fully available without restriction, with rare exception (please refer to the Data Availability Statement in the manuscript PDF file). The data should be provided as part of the manuscript or its supporting information, or deposited to a public repository. For example, in addition to summary statistics, the data points behind means, medians and variance measures should be available. If there are restrictions on publicly sharing data—e.g. participant privacy or use of data from a third party—those must be specified.
Reviewer #1: No
Reviewer #2: Yes
**Answer to reviewers:** The narratives of participants and main verbatims/ quotes supporting the main insights of the study are freely available on the website of AQuAS, the public governmental institution dedicated to evaluating the quality of healthcare and development of decision-making tools/ knowledge transfer.

For further information, please consult:

https://aquas.gencat.cat/web/.content/minisite/aquas/publicacions/2022/estudi_qualitatiu_bona_mort_annexos_aquas2022.pdf

________________________________________
**4. Is the manuscript presented in an intelligible fashion and written in standard English?**

PLOS ONE does not copyedit accepted manuscripts, so the language in submitted articles must be clear, correct, and unambiguous. Any typographical or grammatical errors should be corrected at revision, so please note any specific errors here.
Reviewer #1: Yes
Reviewer #2: Yes

**Answer to reviewers:** A preliminary version of the manuscript (before first submission to PLOS ONE) was revised by a native professional biomedical translator. After including the changes suggested by the editorial team and reviewers, the same translator has revised the new version.

______________________________________
**5. Review Comments to the Author**

Please use the space provided to explain your answers to the questions above. You may also include additional comments for the author, including concerns about dual publication, research ethics, or publication ethics. (Please upload your review as an attachment if it exceeds 20,000 characters)

**Reviewer #1:**
**When looking at the text, I don’t see any obvious red flags. I, however, don’t see the information in the text that I require to be able to make an adequately detailed decision. I was hoping that I would find, attached somewhere, an annex that would allow me to understand exactly what they had done and why.**

**Answer to reviewer 1:** We thank the reviewer for this suggestion and have added a new appendix:

*APPENDIX 1. Techniques and conceptual-methodological approaches used in the qualitative study of a ”good death” in Catalonia (Spain).*

In response to the suggestions of reviewers, the previous Appendix 1 has been replaced.

**I do believe that at the end of my review process I will come to the conclusion that what I’m seeing appears to be sound, but that the initial structure of the article does not adequately anticipate the methodology section, so it requires substantial revision. What I mean by this is that the choices made in the methodology section require information which should be, but is not present in the introduction.**

**Answer to reviewer 1:** In response to this comment we now discuss the rationale of the methodological approach in more detail in the introduction (paragraphs 1 to 3 of the new version on pages 4 and paragraph 1 on page 6).

**I also believe that I will come to the conclusion that the method which they used is not a discourse analysis. As I have often found, I suspect that what I will find, when I have the opportunity to review more detailed information, is that they did a sound thematic content analysis.**

**Answer to reviewer 1:** We carried both a thematic content analysis and a discourse analysis. The interviews and focus group discussion were based on semistructured interviews. After each interview and focus group discussion a pre-analysis was carried out of the transcriptions, and selected verbatims were classified according to the main thematic topics described in table 1 of the manuscript. The main insights that emerged from each interview and groups were discussed by two researchers and further indepth interpretative discourse analysis allowed us to consider the opinions and experiences of patients, relatives and professionals. In the case of professionals, the narratives were considered according to their profiles. To aid in the description of the findings a summary of the main insights is presented. More information regarding the qualitative analysis carried out can be found in the technical report, available in the public repository of our governmental institution.

The study in general included the following main topics:

-Presentation and life story of the interviewee

-Experience and impact of being sick/ getting old (either oneself or a family member)

-Perceptions and meaning of a good death

-Projecting what a good death is

-Resources needed for achieving a good death

We presented participants with images from nature. The participant chose one that they thought would represent a good death. They were also asked about the barriers and facilators for achieving a good death and about the unmet needs in the health system. In the case of professionals, we asked about the aspects they thought could be modified to improve the end of life and facilitate a good death. The perceptions of patients/people needing chronic or palliative care, the elderly, close family members or healthcare professionals were analysed both separately and globally.

**It is a constant frustration of mine that authors in my field, feel, properly, compelled to claim that they are doing analysis methods which are far more complex than that either possible or necessary for their purposes.**

**Answer to reviewer 1:** Three theoretical-methodological perspectives were included in the present qualitative study to broaden our understanding of the meaning of a good death, of barriers and facilitators for achieving it, and also unmet needs. The present paper focused on the the concept of a good death; other findings of the broader study can be consulted elsewhere.

The combination of an ethnographical perspective requiring a more naturalístic and less interpretative analysis with a more hermeneutical approach was carried out in the case of in depth interviews with patients and families. In the case of focus group discussions with professionals, a phenomenological approach was applied. The dynamics of the focus groups included a presentation of the main insights provided by patients and relatives in the study and debate/discussion. The analysis included a discourse analysis with a combination of different degrees of interpretation. The consideration of previously published studies and the discussion of findings with an independent advisory committee aided the validation of the main insights and the verbatims, and gave support to the findings and conclusions made.

The main goal was to understand what a good death is, taking into consideration multiple participant profiles. This approach facilitates triangulation and enriches the analysis and validation of the findings.

**I will find that the article failed to follow the expectations of annotation for transparent inquiry, which means that I do not understand the logic which they have used in sampling quotations for presentation in their text.**

**Answer to reviewer 1:** We agree with the reviewer, and now include additional quotations in the appendix of the new version of the manuscript. We have deleted the old appendix 1, and added new quotes that corroborate more clearly the main findings presented in the paper. We have included more details on the differences according to participant profiles in appendix 2A and 3.

Due to limitations of space and the specific goals defined in this manuscript, we chose to select the main common elements of a projected good death in global terms.

**I will also find that they failed to remain consistent in their recognition that the object, good death, would be understood very differently by their respondents. What this means, in consequence, is that rather than present, a set of scenarios at the end, each some sort of idealized representation selected for centrality.**

**Answer to reviewer 1:** We agree to some extent with the reviewer. As mentioned in the first paragraphs, the answer to the question “is the concept of a good death universal?” is “no”; it is a subjective concept, and the insights gained from the discourses of participants suggest that it should be defined as “dying in the way each individual person wants”. As in other published studies, and to aid the understanding of an abstract, subjective concept, we identified certain common elements, which were then revised and discussed further by the research team and the external independent steering group. Barriers and facilitators and unmet needs were also recorded and described elsewhere, and help to understand what the public health system can do to improve end-of-life care and planning.

The new appendix 3 includes further findings regarding core elements of a good death for patients, relatives and professionals, as well as a table comparing core elements in the present study with those of previous studies that have applied qualitative or quantiative methodological approaches (appendix 4).

**What they have done is present a list of common elements across respondents that are chosen more for commonality than salience. This sort of presentation of an intersection of subjective constructions is a common strategy that is used which suffers the unfortunate characteristic of failing to remember that each of the construction is considered may fail horribly when shorn of attributes specific to that single construction.
Answer to reviewer 1:** as mentioned above, for reasons of space, the previous version of the manuscrit described a single global construction. It is already known that the Institute of Medicine (IOM) and some studies of reference on “good death” discuss the issue of subjectivity in these complex concepts. The same main insight was found in the present study (please see page 13, last paragraph). Nevertheless, to gain more common understanding and to be more specific, the strategy proposing the most common elements seems valid and aids comparison with other studies already published in other populations. The most common and universal element in both our study and others is “comfort and placidity” – the ideal feeling of not suffering or being in pain during the process of dying. In this regard, certain elements described in recent systematic reviews of the topic are included in our study as barriers to or facilitators of a good death.

**At the end, what I think I will find is that the article does an interesting examination of stakeholders understandings of a very complex and subjectively constructed construct, the good death.**

**Answer to reviewer 1:** Thank you for this appreciation. One of the goals of this project was to gather together the points of view of the main stakeholders involved in the process of dying and healthcare, namely patients with complex or palliative care needs, older people, family members and health and social care professionals.

**Reviewer #2:**

**The manuscript would benefit from language editing. In some cases, the language needs to be checked to suit the standard language used in research.
Answer to reviewer 2:** The new version of the manuscript has been improved thanks to the suggestions from the reviewers, and it has been revised by a native professional English biomedical translator.

Extra suggestions for the manuscript;

In the introduction section;

**- The following line; 40, 44 and 45 seem to imply that the author is studying people who wish to die at home and people’s experiences using health care services. In the rest of the manuscript, this is not the case. The author may wish to revise the above lines to reflect the specific problem that you are interrogating; ie the meaning of a good death.**
**Answer to reviewer 2:** We agree with the reviewer. The qualitative study included several goals; due to questions of space and the scoping of the present manuscript for PLOS ONE, the emphasis was placed on the perception, meaning and projection of what a good death represents for participants. The paragraph in the introduction section has been rewritten to clarify the rationale being applied.

Most studies available on good death have included the perspectives of family members and professionals, not people with palliative or complex care needs. Information on the preferences of where, how and with whom people want to die (for example, at home, in a hospital, in a residential care setting, in a hospice) is still limited in Spain, and in particular in Catalonia.

In the methods section we now add that all interviews with patients, people over 65 years old and family members were carried out in their home settings, with the exception of two which were conducted in residential care facilities.

In the discussion we now add that an important facilitator for achieving a good death was the preference of the place of death [main insights described elsewhere; some of the participants’ preferences can be found in Appendix 5]. Two main preferences emerged from their testimonies: the ideal of dying at home as a familiar, intimate, comfortable space, or in the safe, secure, clean space of the hospital. As the new generations are not used to being in contact with death, participants who might initially choose to die at home may also consider the idea of dying in a hospital or residence to spare their family members the memory of their dying at home and the associations that this may produce. However, “intimacy” is an important issue: many express the wish to die without interference, extraneous noise, or people who are not from their family or professionals involved in their dying process [Main results on page 16, first paragraph and Appendix 2B “Intimacy”].

**- Line 69 seem to be incomplete**

**Answer to reviewer 2:** Thank you. This sentence in the first paragraph of the introduction has now been completed.

In the methodology section, please consider the following;

**- The word both in line 85 seem to be inappropriate since the data sources are 3 not 2.
Answer to reviewer 2:** Two techniques were applied: in depth interviews with patients and family membres, and focus group discussions with professionals. But the reviewer is right that there were three types of stakeholder. and this point has been modified in the revised version of the manuscript.

Applying an ethnographic and phenomenological perspective, a two-phase qualitative study was carried out including two qualitative techniques, in-depth interviews with patients, families, and discussion groups with professionals.[**13,14**] The data from the three sources of information were triangulated in order to describe the experiences and opinions of key stakeholders regarding the phenomenon of a good death and its common elements (see topics in **table 1**).

**- The word intentional could be replaced with purposive in reference to the sampling procedure, if that is what the author wanted to mean. Check like 104 , 147 etc
Answer to reviewer 2:** this change has been made (page 8, 3rd paragraph and page 9, last paragraph).

**- The author mentions that they excluded people did not have ‘powerful’ internet connection. The reader would be interested in knowing how they identified these- 164
Answer to reviewer 2:** in this case, none of the patients, elderly and family members were interviewed online; in the case of focus group discussions, participants needed to have an internet connection with enough power to be able to follow a discussion lasting 2½ to 3 hours. The last sentence on the sampling methods of focus group discussions on page 9 has been rewritten.

**- Throughout the manuscript, the author talks about discussion groups and not Focus Group Discussions. Is that intentional?**

**Answer to reviewer 2:** in Spain, there is a slight difference between discussion groups and focus groups. The overall project of which this paper constitutes a part has a broader scope than is reflected here, including a debate on facilitators and barriers and proposals for action to address unmet needs. The intention was to mix profiles of healthcare professionals attending people at the end of their lives, their famílies, clinical managers, planners and also professionals working in patient/family associations. We agree with the reviewer that this qualitative technique can be called “focus group discussion”. This term is now used throughout the manuscript (abstract, ethics statement, letter to editor, authors’ contributions, abstract in the main manuscript materials and methods and table 1, and results section).

In the results section, please consider the following;
**- In line 221, the author clearly shows that participants considered a good death in relation to a good life. One cannot have a good death unless they had a good life. It would be important for the author to explain what a good life was from the perspective of the study participants**.

**Answer to reviewer 2:** This is the case for some participants in our qualitative study. We have added additional insights and quotes to support this finding in appendix 2B (Appendix documents).

**-In the conclusion, the author introduces a new concept of ‘red lines’ in relation to what participants refer to as unbearable suffering. However, it is important that the author introduces these concepts earlier in the manuscript to avoid new information in the conclusion section of the manuscript.**

**Answer to reviewer 2:** Due to the suggestions that we should provide further information added in the main manuscript and appendix, we have removed this point for reasons of space. Priority is now given to the core elements of what a good death is as described by the participants.

_______________________________________
6. **PLOS authors have the option to publish the peer review history of their article (what does this mean?). If published, this will include your full peer review and any attached files.**
If you choose “no”, your identity will remain anonymous but your review may still be made public.
Reviewer #1: **Yes:** Peter tamas

Reviewer #2: No

[NOTE: If reviewer comments were submitted as an attachment file, they will be attached to this email and accessible via the submission site. Please log into your account, locate the manuscript record, and check for the action link "View Attachments". If this link does not appear, there are no attachment files.]

While revising your submission, please upload your figure files to the Preflight Analysis and Conversion Engine (PACE) digital diagnostic tool, <https://pacev2.apexcovantage.com/>. PACE helps ensure that figures meet PLOS requirements. To use PACE, you must first register as a user. Registration is free. Then, login and navigate to the UPLOAD tab, where you will find detailed instructions on how to use the tool. If you encounter any issues or have any questions when using PACE, please email PLOS at [figures@plos.org](mailto:figures@plos.org). Please note that Supporting Information files do not need this step.
