# Supplementary material for: Defining a good death: Perspectives of patients, relatives, and health care professionals in the Catalan context—A qualitative study
Source: PLoS One. 2024 Nov 27;19(11):e0312426. doi: 10.1371/journal.pone.0312426 (PMC11602040; doi:10.1371/journal.pone.0312426)
Supplement: Attachment — Submitted filename: plos1 nov 2023 good death revision.docx [file pone.0312426.s007.docx]

1. Is the manuscript technically sound, and do the data support the conclusions?

Reviewer #1: Partly
Reviewer #2: Partly
________________________________________
2. Has the statistical analysis been performed appropriately and rigorously?
Reviewer #1: I Don't Know
Reviewer #2: N/A
________________________________________
3. Have the authors made all data underlying the findings in their manuscript fully available?

The PLOS Data policy requires authors to make all data underlying the findings described in their manuscript fully available without restriction, with rare exception (please refer to the Data Availability Statement in the manuscript PDF file). The data should be provided as part of the manuscript or its supporting information, or deposited to a public repository. For example, in addition to summary statistics, the data points behind means, medians and variance measures should be available. If there are restrictions on publicly sharing data—e.g. participant privacy or use of data from a third party—those must be specified.
Reviewer #1: No
Reviewer #2: Yes

Comment on revision: the PDF does not meet my understanding of the requirements for either archiving or data. The data are not in an archive, the relationship between the data found and the recording is uncertain, there are no meta-data and there is no qualitative equivalent to the R script I would expect to find with archived data

________________________________________
4. Is the manuscript presented in an intelligible fashion and written in standard English?

PLOS ONE does not copyedit accepted manuscripts, so the language in submitted articles must be clear, correct, and unambiguous. Any typographical or grammatical errors should be corrected at revision, so please note any specific errors here.
Reviewer #1: Yes
Reviewer #2: Yes
________________________________________
5. Review Comments to the Author

Please use the space provided to explain your answers to the questions above. You may also include additional comments for the author, including concerns about dual publication, research ethics, or publication ethics. (Please upload your review as an attachment if it exceeds 20,000 characters)

Reviewer #1:
When looking at the text, I don’t see any obvious red flags. I, however, don’t see the information in the text that I require to be able to make an adequately detailed decision. I was hoping that I would find, attached somewhere, an annex that would allow me to understand exactly what they had done and why.

- Comment on revision: The level of documentation of the methodology and methods remains inadequate.

I do believe that at the end of my review process I will come to the conclusion that what I’m seeing appears to be sound, but that the initial structure of the article does not adequately anticipate the methodology section, so it requires substantial revision.

- Comment on revision: the changes made have degraded my assessment of the rigor of analysis.

What I mean by this is that the choices made in the methodology section require information which should be, but is not present in the introduction.

- Comment on revision: Much of the information required to understand the logic behind selection of methods and the details of their use remain missing.

I also believe that I will come to the conclusion that the method which they used is not a discourse analysis.

- Comment on revision: I still lack the information required to determine whether the analysis conducted qualifies as a discourse analysis. For that I require a defensible definition followed by analytic practice that clearly matches that definition.

As I have often found, I suspect that what I will find, when I have the opportunity to review more detailed information, is that they did a sound thematic contact analysis. It is a constant frustration of mine that authors in my field, feel, properly, compelled to claim that they are doing analysis methods which are far more complex than that either possible or necessary for their purposes.

- Comment on revision: the information provided did not improve insight into the steps by which analysis was undertaken. For example, the terms ‘vertical and then horizontal reading’ do not mean much without both a definition and justification. This journal is read by a wide diversity of readers. They can not be expected to know what these are nor whether they are appropriate. This information must be provided by the authors.

I will find that the article failed to follow the expectations of annotation for transparent inquiry, which means that I do not understand the logic which they have used in sampling quotations for presentation in their text.

- Comment on revision: the authors did not improve the transparency through mechanisms such as ATI.

I will also find that they failed to remain consistent in their recognition that the object, good death, would be understood very differently by their respondents. What this means, in consequence, is that rather than present, a set of scenarios at the end, each some sort of idealized representation selected for centrality. What they have done is present a list of common elements across respondents that are chosen more for commonality than salience. This sort of presentation of an intersection of subjective constructions is a common strategy that is used which suffers the unfortunate characteristic of failing to remember that each of the construction is considered may fail horribly when shorn of attributes specific to that single construction.

- Comment on revision: The authors seem to flip-flop between accepting the possibility of a heterogeneity of mutually incommensurate constructions of ‘the good death’ and the policy convenience of a determinate list of identically understood (and therefore, somehow, important) themes.

Comments made on text

ATLAS.ti Report

plos1 review july 22

Quotations grouped by Documents

Report created by peter tamas on 10 Nov 2023

# 1 PONE-D-23-14275_R1_reviewer.pdf

31 Quotations:

## 1:1 p 19 in PONE-D-23-14275_R1_reviewer.pdf

Content:

We applied an ethnographic

81 perspective, approaching people in their natural and everyday environment and carrying out

82 in-depth interviews in their homes.

Comment:

Semi structured interviews in the home of respondents is not enough to suggest that the research was ethnographic. Claims of this category requires first a definition and then a discussion of practice that demonstrates how the project met the requirements of the definition. Most definitions of ethnographic require participant observation. If you are more specific, however, and suggest that you conducted ethnographic interviews…that might be defensible.

## 1:2 p 19 in PONE-D-23-14275_R1_reviewer.pdf

Content:

This approach helped to generate an environment of trust

83 and a better understanding of the situation of the participants as they expressed their

84 experiences, needs and opinions on what a “good death” means.

Comment:

Why does this result? what are the mechanisms by which it is produced? and what are the ethical implications of manipulating the circumstances of vulnerable respondents such that they are inclined to trust?

## 1:3 p 19 in PONE-D-23-14275_R1_reviewer.pdf

Content:

in order to be able to compare the common and differential elements in our study and in

87 other previously published studies. For this reason, a hermeneutic phenomenological

88 perspective was also applied combining the perspectives of patients and family members

89 with those of professionals in the health system

Comment:

I hope to see more detail of this. You state ‘for this reason’ but I am not clear what the reason is nor why this approach, whatever it is, is appropriate.

## 1:4 p 19 in PONE-D-23-14275_R1_reviewer.pdf

Content:

Given the complexity and the subjective nature of the topic, the hope was that this

combination of techniques, approaches and informants would allow a deeper understanding of the meaning given to a “good death” in Catalonia.

Comment:

Here you note ‘complexity’ and ‘subjective nature’ but then you say ‘the meaning given to a ‘good death’’…not ‘the meanings given to a good death’. How do you support the assumption of a single narrative given that it is complex and subjective?

## 1:5 p 19 in PONE-D-23-14275_R1_reviewer.pdf

Content:

In this article, we present the results of the study and describe the conceptualizations made by the different key actors (patients, relatives and professionals) about what a “good death” is and about the common elements that represent it.

Comment:

What theory guides you to privilege commonality?

## 1:6 p 20 in PONE-D-23-14275_R1_reviewer.pdf

Content:

Applying an ethnographic and phenomenological perspective, a

Comment:

I need to know what these are before I can locate what you have done. Sometimes this is done through citation to detailed discussions and others through explicit discussion. Here there is neither.

## 1:7 p 20 in PONE-D-23-14275_R1_reviewer.pdf

Content:

a two-phase qualitative study

106 was carried out including two qualitative techniques, in-depth interviews with patients and

107 families and focus group discussions with professionals

Comment:

Why did you choose these methods?

## 1:8 p 20 in PONE-D-23-14275_R1_reviewer.pdf

Content:

The data from three sources

108 of information were triangulated in order to describe the experiences and opinions of these

109 key stakeholders regarding the phenomenon of a good death and its common elements (see

110 topics in table 1).

Comment:

Triangulation is generally used with the background assumption of convergence which is not compatible with accepting that realities are constructed…which is a pretty common notion in phenomenological studies. What assumptions guided your use of triangulation?

## 1:9 p 20 in PONE-D-23-14275_R1_reviewer.pdf

Content:

In phase 1, in-depth face-to-face ethnographic interviews were conducted with patients and

117 family members.

Comment:

‘The ethnographic interview’ by Spradley??

## 1:10 p 20 in PONE-D-23-14275_R1_reviewer.pdf

Content:

were carried

118 out by a senior qualitative researcher using a predefined script

Comment:

Why was it important that the interviewer be senior, what design principles shaped the script and how is a predefined script compatible with the ‘ethnographic interview’

## 1:11 p 20 in PONE-D-23-14275_R1_reviewer.pdf

Content:

all the interviews were audiotaped for transcription

Comment:

Which approach to transcription was used? e.g. just words? Intonation? silences? Non verbals?

## 1:12 p 21 in PONE-D-23-14275_R1_reviewer.pdf

Content:

The main insights were revised by the coordinating researcher and

121 then by the steering group.

Comment:

What criteria were used to identify portions of text as a ‘main insight’? What process was used to come up with those criteria? What justifies involvement of these actors?

## 1:13 p 21 in PONE-D-23-14275_R1_reviewer.pdf

Content:

The sampling was

128 stratified and purposive, in an attempt to obtain a wide range of discourses.

Comment:

Purposive sampling for heterogeneity is defensible…but not defended. What was the theory that guided selection of the variables on which you stretched the population in order to identify probable maximal heterogeneity?

## 1:14 p 21 in PONE-D-23-14275_R1_reviewer.pdf

Content:

For convenience,

129 however, people who were available and willing to participate in the study were pre-selected.

Comment:

…and this pretty much destroys the purposive nature of the sample as I truly don’t know anything about these folks. You get left with a convenience sample that does not support generalisation.

## 1:15 p 22 in PONE-D-23-14275_R1_reviewer.pdf

Content:

The theoretical sample was purposive and stratified by four segments

171 according to their professional background.

Comment:

Why was their professional background relevant?

## 1:16 p 23 in PONE-D-23-14275_R1_reviewer.pdf

Content:

Patients’ and professionals’ residence (defined as urban or rural) and the health region in

196 Catalonia in which they lived or worked were also recorded.

Comment:

These are descriptive variables. Why were these found to be relevant?

## 1:17 p 23 in PONE-D-23-14275_R1_reviewer.pdf

Content:

During data mining, relevant statements were selected to reinforce the main insights

Comment:

It is not at all clear what is meant by this statement. Data mining is a procedure normally associated with quantitative exploration of very large sets of data (either numeric or narrative). The second part seems to suggest that data were retained when they confirmed initial biases. If so, that would be concerning as it smells like cherry picking.

## 1:18 p 23 in PONE-D-23-14275_R1_reviewer.pdf

Content:

A

199 qualitative content and discourse analysis

Comment:

While qualitative content analysis has been well discussed over the years, the diversity of approaches bearing that label demands near step by step explanation. As for qualitative discourse analysis, the diversity of notions of discourse has long been well documented so the term, as used here, has no explanatory value.

## 1:19 pp 23 – 24 in PONE-D-23-14275_R1_reviewer.pdf

Content:

A

199 qualitative content and discourse analysis was conducted for each sample segment and

[10]

200 globally for each relevant topic.[15]

Comment:

It is not clear how citation to Mays N, Pope C. Qualitative research: rigor and qualitative research. BMJ. 1995;

557 311:109. doi: https://doi.org/10.1136/bmj.311.6997.109 supports the claim here. The one standard about which there is complete agreement across the diversity of approaches to analysis is that of transparency. It is on this expectation that I find the paper particularly deficient. This silence, coupled with the use of terms that are socially accepted but empirically vague concerns me deeply.

## 1:20 p 24 in PONE-D-23-14275_R1_reviewer.pdf

Content:

The transcriptions were read vertically, and the discourse

201 was broken down into different sub-categories and then read horizontally.

Comment:

Readers can not be expected to understand what vertical and horizontal reading are.

## 1:21 p 24 in PONE-D-23-14275_R1_reviewer.pdf

Content:

An inductive

202 process of organizing the qualitative data was applied to detect patterns of behavioral and

203 attitudinal responses

Comment:

The presumption here is that meaning is latent in the text. That epistemic stance requires justification.

## 1:22 p 24 in PONE-D-23-14275_R1_reviewer.pdf

Content:

The results of the in-depth ethnographic interviews (phase 1) were

204 contrasted with the results of the focus group discussions (phase 2), and were then

205 discussed by the core research team and subsequently with the steering group to confirm

206 their coherence and robustness.

Comment:

Comparison is only admissible after discussion of their equivalence. Comparison absent understanding of the grounds that support that act is not interpretable.

It is not clear what discussion within the research team was expected to produce nor is there theory presented anywhere that would support that understanding.

Coherence and robustness are epistemic standards whose operationalisation and expectations vary by context and theory. This is not discussed.

## 1:23 p 24 in PONE-D-23-14275_R1_reviewer.pdf

Content:

The results of the present qualitative study were

207 triangulated with those of related publications identified in an ad hoc literature review for the

208 validation of the main insights.

Comment:

It is not clear what triangulation is supposed to be in this study.

## 1:24 p 26 in PONE-D-23-14275_R1_reviewer.pdf

Content:

Common elements of the perception of a “good death”

Comment:

It is not clear why prevalence of an opinion generated by unequal measures (i.e. interviews and/or focus groups) within a convenience sample matters.

## 1:25 p 27 in PONE-D-23-14275_R1_reviewer.pdf

Content:

Of these eight elements, the most universal and frequently present was comfort and

264 placidity, understood as the absolute absence of physical suffering, such as suffocation and

265 pain, and mental suffering, such as anxiety and distress.

Comment:

Here again the authors are suggesting that prevalence matters. Prevalence only matters when there is a known relationship between a sample and a population and when data are the result of a direct prompt. When asked about what matters, people report what itches which only partly coincides with what is.

## 1:26 p 33 in PONE-D-23-14275_R1_reviewer.pdf

Content:

This study has explored the idea of what a good death is for patients, families and

429 professionals and has identified a set of common elements that mainly reflect the need to

430 help people to die in accordance with their wishes.

Comment:

It is not clear that the theory supporting the study supports the notion that commonality matters.

## 1:27 p 33 in PONE-D-23-14275_R1_reviewer.pdf

Content:

In general, our study corroborates the results of the previous literature, in so far as there is

441 no single universal definition of a “good death”.[1-5]

Comment:

The statement in the previous paragraph that privileged commonality does not appear to fit well with this agreement that what constitutes a good death is specific.

## 1:28 p 33 in PONE-D-23-14275_R1_reviewer.pdf

Content:

To offer an operational definition that

442 might lead to improvements in the health system and elsewhere, we propose a series of

443 common elements which we have identified as salient and which, we believe, can help to

444 make abstract and subjective preferences more concrete.

Comment:

It is not clear that inconsistent measurement within a convenience sample is adequate to support the sort of population level predictive claims normally thought necessary to back policy recommendations. There are, of course, all sorts of other ways to justify such prescriptive claims...but the authors are silent on the argument necessary to take that step so it is reasonable to default to the expectations of Neo-positivist science in a complicated but well understood context.

## 1:29 p 34 in PONE-D-23-14275_R1_reviewer.pdf

Content:

In the context of the widespread reticence in society to talk openly about death,

Comment:

It is very late in the report to be introducing description of relevant aspects of the context of the study. These aspects are normally introduced earlier in order to inform the design of data collection. In this case, for example, ‘taboo’ would be expected to inform response rates…which would have an impact on recruitment practices for the study.

## 1:30 p 35 in PONE-D-23-14275_R1_reviewer.pdf

Content:

The main limitation of this study is the possible selection bias.

Comment:

This limitation is very well noted. It is not clear, however, that the authors respected the consequences of that limitation. Selection bias would produce non-representative results that may compromise population level conclusions which would have an impact on justification required for policy recommendations. That chain of implications does not appear to have been respected.

## 1:31 p 35 in PONE-D-23-14275_R1_reviewer.pdf

Content:

As far as achieving a single universal and unequivocal definition of a "good death" is

492 concerned, the study confirms that people's perceptions are subjective and unique, just as

493 they would be regarding a definition of what constitutes a "good life". Despite this subjective

494 aspect, eight common elements emerged from the interviews.

Comment:

The assertion of specificity followed by the construction ‘despite this subjective aspect’ leads me to worry that the authors are grasping for actionable objective universals. This combination suggests a fundamental incoherence in the design of the research.
