# Supplementary material for: Defining a good death: Perspectives of patients, relatives, and health care professionals in the Catalan context—A qualitative study
Source: PLoS One. 2024 Nov 27;19(11):e0312426. doi: 10.1371/journal.pone.0312426 (PMC11602040; doi:10.1371/journal.pone.0312426)
Supplement: Attachment — Submitted filename: Answers to editors and reviewers.docx [file pone.0312426.s008.docx]

**PONE-D-23-14275R2**

**TITLE:** Defining a Good Death: Perspectives of Patients, relatives, and Health Care Professionals in the Catalan Context—A Qualitative Study

PLOS ONE

Dear Dr. Serra-Sutton,
Thank you for submitting your manuscript to PLOS ONE. After careful consideration, we feel that it has merit but does not fully meet PLOS ONE’s publication criteria as it currently stands. Therefore, we invite you to submit a revised version of the manuscript that addresses the points raised during the review process.

Please see below under the section additional Editor's comments, the summary of the issues that you need to address
==============================
Please submit your revised manuscript by Jul 17 2024 11:59PM. If you will need more time than this to complete your revisions, please reply to this message or contact the journal office at [plosone@plos.org](mailto:plosone@plos.org). When you're ready to submit your revision, log on to <https://www.editorialmanager.com/pone/> and select the 'Submissions Needing Revision' folder to locate your manuscript file.

Please include the following items when submitting your revised manuscript:

- A rebuttal letter that responds to each point raised by the academic editor and reviewer(s). You should upload this letter as a separate file labeled 'Response to Reviewers'.
- A marked-up copy of your manuscript that highlights changes made to the original version. You should upload this as a separate file labeled 'Revised Manuscript with Track Changes'.
- An unmarked version of your revised paper without tracked changes. You should upload this as a separate file labeled 'Manuscript'.

If you would like to make changes to your financial disclosure, please include your updated statement in your cover letter. Guidelines for resubmitting your figure files are available below the reviewer comments at the end of this letter.
If applicable, we recommend that you deposit your laboratory protocols in protocols.io to enhance the reproducibility of your results. Protocols.io assigns your protocol its own identifier (DOI) so that it can be cited independently in the future. For instructions see: <https://journals.plos.org/plosone/s/submission-guidelines#loc-laboratory-protocols>. Additionally, PLOS ONE offers an option for publishing peer-reviewed Lab Protocol articles, which describe protocols hosted on protocols.io. Read more information on sharing protocols at <https://plos.org/protocols?utm_medium=editorial-email&utm_source=authorletters&utm_campaign=protocols>.

We look forward to receiving your revised manuscript.

Kind regards,

Martin Mbonye
Academic Editor
PLOS ONE

Journal Requirements:

Please review your **reference list** **to ensure that it is complete and correct**. If you have cited papers that have been retracted, please include the rationale for doing so in the manuscript text, or remove these references and replace them with relevant current references. Any changes to the reference list should be mentioned in the rebuttal letter that accompanies your revised manuscript. If you need to cite a retracted article, indicate the article’s retracted status in the References list and also include a citation and full reference for the retraction notice.

**Answer to the editorial team:**

The bibliographic reference list has been thoroughly revised and updated. As a result of further methodological revisions, additional references have been included**.** Please refer to references 12, 13, 15 and 16.

Some references have been renumbered to improve clarity, following suggestions from the reviewers and the editorial team. Thus, changes have been made in some paragraphs within the introduction and discussion sections. Please see references 1, 2 (before 11, 12), and also 19 to 22 (before 7 to 10 and 19).

Moreover, the references to the corresponding additional appendix materials (new and renumbered) have been accurately referenced in the main manuscript.

Additional Editor Comments:

Dear Author,
Thank you very much for your patience with this paper. It is almost there but there are a few things that were suggested by the reviewer that I feel still need your attention as well as a few things. Below are the areas that you should look at and address before we can proceed.

**Answer to the editorial team:**

Thank you very much for your kind words and your patience with us throughout this process. Our primary focus is on producing technical reports and facilitating knowledge transfer products. In the context of the present study and under the observatory of death, we have produced grey literature and educational materials for different audiences to understand what a good death is in Catalonia, Please refer to:

<https://observatorisalut.gencat.cat/ca/observatori_mort/estudi-de-la-bona-mort/>

<https://observatorisalut.gencat.cat/ca/observatori_mort/>

This scientific manuscript is our first submission to PLOS ONE based on our qualitative study. Following the feedback from reviewers and the editorial team, we have refined our terminology regarding techniques and classifications of theoretical, methodological, and analytical approaches. We also conducted a thorough review of the relevant academic literature on qualitative research in healthcare to strengthen our work.

The review process of the manuscript has improved our knowledge, clarity, and confidence in presenting what we hope is a meaningful and robust study for publication in your journal.

1. Under materials and Methods: The reviewer was not satisfied that you had addressed in your revision of the methods section. The reviewer suggested that you give more details and justify the selection of the approach you use. I suggest that instead of pointing the reader to another location for details that instead you carefully read the comments on this particular issue and add more details.

**NEW answer to editorial team (September 2024):** We have added more detail and rationale of the methods, paradigms, and theoretical-methodological approaches in the introduction section. Additionally, we have added a NEW Appendix 1 to provide further context, research questions, and justification for their application.

Please see below the new answers to reviewers 1 and 2. We have retained the previous answers to the editorial team and the reviewers made in May 2024 for reference, ensuring clarity of both the issues addressed and the changes implemented to improve the manuscript.

2. Under sampling: Please be clear on which sample was selected under each of your sampling procedures. For example, who was purposively selected and who was selected by convenience sampling and why?

**NEW answer to editorial team (September 2024):** We have now improved our explanation of the sampling procedures and the type of sampling design in the case of patients and families (relatives) for semi-structured interviews and professionals in the focus group discussions. It is also described in a structured manner with bibliographic references in the new Appendix 1. Please see below the new answers to reviewers 1 and 2. We have retained the previous answers to the editorial team and the reviewers made in May 2024 for reference ensuring clarity of both the issues addressed and the changes implemented to improve the manuscript.

3. Under materials: Say something on the method used of Phenomenology in order to satisfy the reviewer's misgivings about clarity of methods. Also show why it was the most suited for answering your research question for readers unfamiliar with the concept.

**NEW answer to editorial team (September 2024):** We have added more rationale to justify the theoretical-methodological approaches followed as mentioned, offering more clarity and coherence in the introduction, methods section, and also Appendix 1.

Please see below the NEW answers to reviewers 1 and 2. Please see below the new answers to reviewers 1 and 2. We have retained the previous answers to the editorial team and the reviewers made in May 2024 for reference ensuring clarity of both the issues addressed and the changes implemented to improve the manuscript.

4. I suggest that you include a section of the setting so that the non-Spanish readers appreciate the (rural and ethnic) context. You mention Catalonia but it may not be universally known

**NEW answer to the editorial team (September 2024):** We have added more information of the context where the qualitative study took place in Appendix 1 (points 1 and 5).

5. Under data analysis: Please respond to the reviewer's comment on finding a definition of discourse analysis and how you applied the discourse analysis based on your definition

**NEW answer to the editorial team (September 2024):** We have added more rationale to justify the definition of both the “thematic content analysis” and the “discourse analysis” in the Methods section ("Analysis" subsection) and Appendix 1 (points 17 and 18). Please see below the new answers to reviewers 1 and 2. We have left the previous answers to the editorial team and the reviewers made in May 2024 for reference ensuring clarity of both the issues addressed and the changes implemented to improve the manuscript.

6. The reviewer was not satisfied that your use of the terms vertical and horizontal under the analysis section added much value. So I suggest that you explain them clearly or replace them with more orthodox terms

**NEW answer to editorial team (September 2024):** We improved the clarity of the analytical section in the Methods and the Appendix 1 (points 17 and 18). We modified the terms vertical and horizontal reading to match common terminology in published academic qualitative research in healthcare. The revised manuscript also includes additional bibliographic references to support these updates.

7. You were asked to explain transparancy in application of participants quotes. I think a way around this would be to say something in the methods section indicating your use of quotes. Read any document you can find on annotation for transparency inquiry (ATI) for guidance

**NEW answer to the editorial team (September 2024):** We have added in the method section (“Analysis” subsection), that vívid quotations were selected to reinforce the themes and insights presented, adding short information of the profile of participant to understand a little of their context.

8. Lastly, there are still a number of gramatical errors and typos in the revised version. Please use an English speaker to help with the editor after you are done
**NEW answer to editorial team (September 2024):** A professional English speaker and a biomedical scientific writer and translators have revised this last version of the manuscript that is now being sent for consideration to be published in PLOS ONE.

[Note: HTML markup is below. Please do not edit.]

Reviewers' comments: These have been summarised above


[NOTE: If reviewer comments were submitted as an attachment file, they will be attached to this email and accessible via the submission site. Please log into your account, locate the manuscript record, and check for the action link "View Attachments". If this link does not appear, there are no attachment files.]

While revising your submission, please upload your figure files to the Preflight Analysis and Conversion Engine (PACE) digital diagnostic tool, <https://pacev2.apexcovantage.com/>. PACE helps ensure that figures meet PLOS requirements. To use PACE, you must first register as a user. Registration is free. Then, login and navigate to the UPLOAD tab, where you will find detailed instructions on how to use the tool. If you encounter any issues or have any questions when using PACE, please email PLOS at [figures@plos.org](mailto:figures@plos.org). Please note that Supporting Information files do not need this step.

******************

**PREVIOUS REQUIREMENTS version sent to PLOS ONE**

**3rd of MAY 2024 UPDATED 30th of SEPTEMBER 2024.**

**Please ensure that your manuscript meets PLOS ONE's style requirements, including those for file naming. The PLOS ONE style templates can be found at**

<https://journals.plos.org/plosone/s/file?id=wjVg/PLOSOne_formatting_sample_main_body.pdf> and

<https://journals.plos.org/plosone/s/file?id=ba62/PLOSOne_formatting_sample_title_authors_affiliations.pdf>

**Previous answer to editorial team (May 2024):** The authors have followed the style requirements of PLOS ONE.

**2. Thank you for stating the following financial disclosure:**

"The study is part of a governmental commissioned project of the General Directorate of Health of the Catalan Health Department from the Government of Catalonia (Spain) in the context of the implementation of the Death Observatory to the Agency for Quality in Healthcare and Assessment of Catalonia (AQuAS)."

At this time, please address the following queries:

a)        **Please clarify the sources of funding (financial or material support) for your study. List the grants or organizations that supported your study, including funding received from our institution.**

**Previous answer to the editorial team (May 2024) :** As mentioned above, the study is part of a commissioned project but did not receive any specific funding or grant for its implementation.

**NEW additional answer to the editorial team (September 2024):** The present qualitative study was carried out under a wider project and in the context of the Death Observatory of Catalonia led by the Agency for Healthcare Quality and Assessment of Catalonia (AQuAS) as a commissioned project of the General Directorate of Health of the Catalan Health Department from the Government of Catalonia (Spain) and its general budget (Good Death Study Code: 0002.LE01.20.037).

b)        **State what role the funders took in the study. If the funders had no role in your study, please state: “The funders had no role in study design, data collection and analysis, decision to publish, or preparation of the manuscript.”**

**Previous answer to the editorial team (May 2024):** The commissioners had no role in study design, data collection or analysis, the decision to publish, or the preparation of this manuscript. The research protocol and preliminary findings were discussed with the commissioners from the Department of Health of Catalonia to increase usefulness and impact for knowledge transfer and decision-making together with the steering committee.

c)        **If any authors received a salary from any of your funders, please state which authors and which funders.**

**NEW additional answer to the editorial team (September 2024):** None of the coauthors received a salary from the funders. The Agency for Healthcare Quality and Assessment of Catalonia (AQuAS) coordinated the qualitative study under a commission, supported by an annual budget from the Health Department. As mentioned, the present study did not receive any specific funding or grants. Lluïsa Fernández Giner received a salary from the Agency for Healthcare Quality and Assessment of Catalonia (AQuAS) as an external and independent consultant paid through a general budget of an internal commissioned project linked to the Death Observatory of Catalonia (Good Death Study Code: 0002.LE01.20.037).

d)        **If you did not receive any funding for this study, please state: “The authors received no specific funding for this work.”**

**Previous answer to the editorial team (May 2024):** The authors received no specific funding for this work.

Please include your amended statements within your cover letter; we will change the online submission form on your behalf.

3. **In your Data Availability statement, you have not specified where the minimal data set underlying the results described in your manuscript can be found.** PLOS defines a study's minimal data set as the underlying data used to reach the conclusions drawn in the manuscript and any additional data required to replicate the reported study findings in their entirety. All PLOS journals require that the minimal data set be made fully available. For more information about our data policy, please see <http://journals.plos.org/plosone/s/data-availability>.

**Previous answer to editorial team (May 2024):** The narratives of participants and verbatims/quotes supporting the main insights of the study are freely available on the website of AQuAS, the public governmental institution dedicated to evaluating the quality of healthcare and the development of decision-making tools/knowledge transfer, particularly regarding this qualitative study.

For further information, please refer to:

https://aquas.gencat.cat/web/.content/minisite/aquas/publicacions/2022/estudi_qualitatiu_bona_mort_annexos_aquas2022.pdf

Upon re-submitting your revised manuscript, please upload your study’s minimal underlying data set as either Supporting Information files or to a stable, public repository and include the relevant URLs, DOIs, or accession numbers within your revised cover letter. For a list of acceptable repositories, please see <http://journals.plos.org/plosone/s/data-availability#loc-recommended-repositories>. Any potentially identifying patient information must be fully anonymized.

Important: If there are ethical or legal restrictions to sharing your data publicly, please explain these restrictions in detail. Please see our guidelines for more information on what we consider unacceptable restrictions to publicly sharing data: <http://journals.plos.org/plosone/s/data-availability#loc-unacceptable-data-access-restrictions>. Note that it is not acceptable for the authors to be the sole named individuals responsible for ensuring data access.

We will update your Data Availability statement to reflect the information you provide in your cover letter.

4. **We note that you have stated that you will provide repository information for your data at acceptance.** Should your manuscript be accepted for publication, we will hold it until you provide the relevant accession numbers or DOIs necessary to access your data. If you wish to make changes to your Data Availability statement, please describe these changes in your cover letter and we will update your Data Availability statement to reflect the information you provide.

**NEW additional answer to the editorial team (September 2024):** Additional materials are available upon request from the study coordinator. Most information for further research can be found on our website, as previously mentioned.

https://aquas.gencat.cat/web/.content/minisite/aquas/publicacions/2022/estudi_qualitatiu_bona_mort_annexos_aquas2022.pdf

<https://observatorisalut.gencat.cat/ca/detalls/article/bona_mort>

<https://observatorisalut.gencat.cat/ca/observatori_mort/>

 5. **Please include captions for your Supporting Information files at the end of your manuscript, and update any in-text citations to match accordingly**. Please see our Supporting Information guidelines for more information: <http://journals.plos.org/plosone/s/supporting-information>.

**Previous answer to editorial team (May 2024):** Captions have now been included in the appendix material.

Reviewers' comments:

Reviewer's Responses to Questions
Comments to the Author

**1. Is the manuscript technically sound, and do the data support the conclusions?**

Reviewer #1: Partly
Reviewer #2: Partly

**Previous answer to the editorial team (May 2024):** We have improved the manuscript by including additional verbatims/quotations that support the main findings and conclusions.
________________________________________

**2. Has the statistical analysis been performed appropriately and rigorously?**
Reviewer #1: I Don't Know
Reviewer #2: N/A

**Previous answer to the editorial team (May 2024):** The project applied a qualitative approach; as no quantitative data were obtained, no statistical analysis was performed.

___________________________________
**3. Have the authors made all data underlying the findings in their manuscript fully available?**

The PLOS Data policy requires authors to make all data underlying the findings described in their manuscript fully available without restriction, with rare exception (please refer to the Data Availability Statement in the manuscript PDF file). The data should be provided as part of the manuscript or its supporting information, or deposited to a public repository. For example, in addition to summary statistics, the data points behind means, medians and variance measures should be available. If there are restrictions on publicly sharing data—e.g. participant privacy or use of data from a third party—those must be specified.
Reviewer #1: No
Reviewer #2: Yes
**Previous answer to the editorial team (May 2024):** The narratives of participants and main verbatims/quotes supporting the main insights of the study are freely available on the website of AQuAS, the public governmental institution dedicated to evaluating the quality of healthcare and the development of decision-making tools/knowledge transfer.

For further information, please refer to:

<https://aquas.gencat.cat/web/.content/minisite/aquas/publicacions/2022/estudi_qualitatiu_bona_mort_annexos_aquas2022.pdf>

<https://observatorisalut.gencat.cat/ca/detalls/article/bona_mort>

<https://observatorisalut.gencat.cat/ca/observatori_mort/>

________________________________________
**4. Is the manuscript presented in an intelligible fashion and written in standard English?**

PLOS ONE does not copyedit accepted manuscripts, so the language in submitted articles must be clear, correct, and unambiguous. Any typographical or grammatical errors should be corrected at revision, so please note any specific errors here.
Reviewer #1: Yes
Reviewer #2: Yes

**Previous answer to editorial team (May 2024):** A preliminary version of the manuscript (before first submission to PLOS ONE) was revised by a native professional biomedical translator. After including the changes suggested by the editorial team and reviewers, the same translator has revised the new version.

**NEW additional answer to the editorial team (September 2024):** A biomedical scientific writer and a native professional biomedical English translator have revised the present version of the manuscript.

______________________________________
**5. Review Comments to the Author**

Please use the space provided to explain your answers to the questions above. You may also include additional comments for the author, including concerns about dual publication, research ethics, or publication ethics. (Please upload your review as an attachment if it exceeds 20,000 characters)

**Reviewer #1:**
**When looking at the text, I don’t see any obvious red flags. I, however, don’t see the information in the text that I require to be able to make an adequately detailed decision. I was hoping that I would find, attached somewhere, an annex that would allow me to understand exactly what they had done and why.**

**NEW answer to Reviewer 1 (September 2024):** A new Appendix 1 *“Conceptual, theoretical and methodological considerations of the qualitative study on good death in Catalonia”* has been defined and added to provide clearer understanding of what was done exactly in the present qualitative study. This information has made the theoretical-methodological approach, goals, research question, techniques, and analytical process more coherent.

*The previous appendix has been removed: Techniques and conceptual-methodological approaches used in the qualitative study of a "good death" in Catalonia (Spain).*

**I do believe that at the end of my review process I will come to the conclusion that what I’m seeing appears to be sound, but that the initial structure of the article does not adequately anticipate the methodology section, so it requires substantial revision. What I mean by this is that the choices made in the methodology section require information which should be, but is not present in the introduction.**

**NEW answer to Reviewer 1 (September 2024):** In response to this suggestion, the introduction section has been improved from the version sent in May 2024 providing clarity and justification for the present qualitative study on good death in Catalonia. It also clarifies its soundness. Please find further mention of the methodological justification in the 3rd and 4th paragraphs of page 7, further rationale of the qualitative study in the introduction section, and its context in the 2nd paragraph of page 8. This information has also been described in a structured manner in the new version of Appendix 1.

**I also believe that I will come to the conclusion that the method which they used is not a discourse analysis. As I have often found, I suspect that what I will find, when I have the opportunity to review more detailed information, is that they did a sound thematic content analysis.**

**NEW answer to Reviewer 1 (September 2024):** We have added more rationale for both the thematic content analysis and the discourse analysis in the Methods section and Analysis subsection in the first paragraphs of page 13. Also, in Appendix 1, together with the steps carried out in the analytical process (points 17, 18, and 19).

**It is a constant frustration of mine that authors in my field, feel, properly, compelled to claim that they are doing analysis methods which are far more complex than that either possible or necessary for their purposes.**

**NEW answer to Reviewer 1 (September 2024):** We have revised in-depth the nomenclature and approaches followed in the literature and also have adjusted them to a proper description. First, by adding the research questions, the correct theoretical-methodological approaches, techniques, samplings, and analyses carried out. The rationale has been added in the Appendix 1 with bibliographic references. We have added, as mentioned before, more rationale about the theoretical approach and research questions in the introduction.

The project is a complex one with a complex topic. The research team triangulated approaches, techniques, participants, and sources of information.

We hope we have adequately addressed the concerns raised by Reviewer 1.

**I will find that the article failed to follow the expectations of annotation for transparent inquiry, which means that I do not understand the logic which they have used in sampling quotations for presentation in their text.**

**Previous answer to Reviewer 1 (May 2024):** We agree with the reviewer, and now we include additional quotations in the appendix of the new version of the manuscript. We have deleted the old Appendix 1 and added new quotes that corroborate more clearly the main findings presented in the paper. We have included more details on the differences according to participant profiles in Appendix 3.

**I will also find that they failed to remain consistent in their recognition that the object, good death, would be understood very differently by their respondents. What this means, in consequence, is that rather than present, a set of scenarios at the end, each some sort of idealized representation selected for centrality.**

**Previous answer to Reviewer 1 (May 2024):** We partially agree with the reviewer. As mentioned in the first paragraphs, the answer to the question “Is the concept of a good death universal?” is “no.” It is a subjective concept, and the insights gained from the narratives of participants suggest that it should be defined as “dying in the way each person wants.” As in other published studies, and to help with the understanding of an abstract, subjective concept, we identified certain common elements, which were then revised and discussed further by the research team and the external independent Steering Group. Barriers and facilitators and unmet needs were also recorded and described as findings of the present project with a wider scope, and help to understand what the public health system can do to improve end-of-life care and planning.

The new Appendix 3 includes further findings regarding what the core elements of a good death are for patients, relatives, and professionals, as well as a table comparing these core elements in the study with those of previous studies that have applied qualitative or quantitative methodological approaches (Appendix 5).

**What they have done is present a list of common elements across respondents that are chosen more for commonality than salience. This sort of presentation of an intersection of subjective constructions is a common strategy that is used which suffers the unfortunate characteristic of failing to remember that each of the construction is considered may fail horribly when shorn of attributes specific to that single construction.
Previous answer to Reviewer 1 (May 2024):** As mentioned above, the previous version of the manuscript presented a single global construction due to space constraints. It is well known that the Institute of Medicine (IOM) and some studies of reference on “good death” discuss the issue of subjectivity in these complex concepts. The same main insight was found in the present study in the Catalan context (Northeast of Spain). Nevertheless, to gain more understanding and facilitate the planning of common actions to improve end-of-life care, proposing the most common elements appears valid and allows for comparisons with other published studies in different populations. The most common and universal element in both our study and others is “comfort and placidity” – the ideal feeling of not suffering or being in pain during the process of dying. In this regard, certain elements described in recent systematic reviews of the topic are included in our study as barriers to or facilitators for achieving this "good death."

**At the end, what I think I will find is that the article does an interesting examination of stakeholders understandings of a very complex and subjectively constructed construct, the good death.**

**Previous answer to Reviewer 1 (May 2024):** Thank you for this appreciation. One of the goals of this project was to gather together the points of view of the main stakeholders involved in the process of dying and healthcare, specifically patients with complex or palliative care needs, older people, relatives, and health and social care professionals.

**Reviewer #2:**

**The manuscript would benefit from language editing. In some cases, the language needs to be checked to suit the standard language used in research.
Previous answer to Reviewer 2 (May 2024):** The new version of the manuscript has been improved thanks to the suggestions from the reviewers, and it has been revised by a native professional English biomedical translator.

Extra suggestions for the manuscript;

In the introduction section;

**- The following line; 40, 44 and 45 seem to imply that the author is studying people who wish to die at home and people’s experiences using health care services. In the rest of the manuscript, this is not the case. The author may wish to revise the above lines to reflect the specific problem that you are interrogating; ie the meaning of a good death.**
**Previous answer to reviewer 2 (May 2024):** We agree with the reviewer. The qualitative study included several goals. Due to questions of space and the scoping of the present manuscript for PLOS ONE, the emphasis was placed on the perception, meaning, and projection of what a good death represents for participants. The paragraph in the introduction section has been rewritten to clarify the rationale being applied.

Most studies available on a good death have included the perspectives of family members and professionals, not people with palliative or complex care needs. Information on the preferences of where, how, and with whom people want to die (for example, at home, in a hospital, in a residential care setting, or in a hospice) is still limited in Spain, and in particular in Catalonia.

In the methods section, we now add that all interviews with patients, people over 65 years old, and family members were carried out in their home settings, except for two which were conducted in residential care facilities.

In the discussion, we have added that an important facilitator for achieving a good death was the preference for the place of death [main insights described elsewhere; some of the participants’ preferences can be found in Appendix 4]. Two main preferences emerged from their testimonies: the ideal of dying at home as a familiar, intimate, comfortable space, or in the safe, secure, clean space of the hospital. As the new generations are not used to being in contact with death, participants who might initially choose to die at home may also consider the idea of dying in a hospital or nursing home to spare their family members the memory of their dying at home and the associations that this may produce. However, “intimacy” is an important issue: many express the wish to die without interferences, extraneous noises, or people who are neither from their family nor professionals involved in their dying process [main results on page 17, third paragraph and Appendix 3B “Intimacy”].

**- Line 69 seem to be incomplete**

**Previous answer to Reviewer 2 (May 2024):** Thank you. This sentence in the first paragraph of the introduction has now been completed.

In the methodology section, please consider the following;

**- The word both in line 85 seem to be inappropriate since the data sources are 3 not 2.
NEW answer to Reviewer 1 (September 2024):** Two techniques were applied: semi-structured in-depth interviews with patients and relatives, and focus group discussions with professionals. But the reviewer is right that there were three types of stakeholders. This point has been modified in the revised version of the manuscript.

By primarily applying a phenomenological perspective along with hermeneutical and socio-constructivist theorethical-methodological approach, we conducted a two-phase qualitative study using two qualitative techniques, semi-structured in-depth interviews with patients, relatives, and focus group discussions with professionals. The data from the three sources of information were triangulated to describe the experiences and opinions of key stakeholders regarding the phenomenon of a good death and its common elements.

**- The word intentional could be replaced with purposive in reference to the sampling procedure, if that is what the author wanted to mean. Check like 104 , 147 etc
Previous answer to Reviewer 2 (May 2024):** This change has been made (page 8, 3rd paragraph, and page 9, last paragraph).

**- The author mentions that they excluded people did not have ‘powerful’ internet connection. The reader would be interested in knowing how they identified these- 164
Previous answer to Reviewer 2 (May 2024):** In this case, none of the patients and relatives were interviewed online. In the case of the focus group discussions, participants needed to have an internet connection with enough power to be able to follow a discussion lasting 2½ to 3 hours. The last sentence on the sampling methods of focus group discussions on page 9 has been rewritten.

**- Throughout the manuscript, the author talks about discussion groups and not Focus Group Discussions. Is that intentional?**

**Previous answer to Reviewer 2 (May 2024):** In Spain, there is a slight difference between discussion groups and focus groups. The overall project of which this paper constitutes a part has a broader scope than the one reflected here, including a debate on facilitators and barriers and proposals for action to address unmet needs. The intention was to mix profiles of healthcare professionals attending people at the end of their lives, their families, clinical managers, planners, and also professionals who work in patient/family associations. We agree with the reviewer that this qualitative technique can be called “focus group discussion”. This term is now used throughout the manuscript (abstract, ethics statement, letter to editor, authors’ contributions, abstract in the main manuscript, materials and methods, Table 1, and results section).

In the results section, please consider the following;
**- In line 221, the author clearly shows that participants considered a good death in relation to a good life. One cannot have a good death unless they had a good life. It would be important for the author to explain what a good life was from the perspective of the study participants**.

**Previous answer to Reviewer 2 (May 2024):** This is the case for some participants in our qualitative study. We have added additional insights and quotes to support this finding in Appendix 3 (appendix documents).

**-In the conclusion, the author introduces a new concept of ‘red lines’ in relation to what participants refer to as unbearable suffering. However, it is important that the author introduces these concepts earlier in the manuscript to avoid new information in the conclusion section of the manuscript.**

**Previous answer to Reviewer 2 (May 2024):** Due to the suggestions that we should provide further information in the main manuscript and appendix, we have removed this point for reasons of space. We have focused our priority on the core elements of what a good death is as described by the participants.
